# Niche dynamics of alien species do not differ among sexual and apomictic flowering plants

**DOI:** 10.1111/nph.13694

**Published:** 2015-10-28

**Authors:** Agnes S. Dellinger, Franz Essl, Diego Hojsgaard, Bernhard Kirchheimer, Simone Klatt, Wayne Dawson, Jan Pergl, Petr Pyšek, Mark van Kleunen, Ewald Weber, Marten Winter, Elvira Hörandl, Stefan Dullinger

**Affiliations:** ^1^ Department of Botany and Biodiversity ResearchUniversity of ViennaRennweg 14Vienna1030Austria; ^2^ Department of Systematics, Biodiversity and Evolution of PlantsGeorg‐August‐University of GöttingenUntere Karspüle 2Göttingen37073Germany; ^3^ EcologyUniversity of KonstanzUniversitätsstrasse 10Konstanz78457Germany; ^4^ Institute of BotanyDepartment of Invasion EcologyThe Czech Academy of SciencesPrůhoniceCZ‐252 43Czech Republic; ^5^ Department of EcologyFaculty of ScienceCharles University in PragueViničná 7CZ‐128 44Prague 2Czech Republic; ^6^ Institute of Biochemistry and BiologyUniversity of PotsdamMaulbeerallee 1Potsdam14469Germany; ^7^ German Centre for Integrative Biodiversity Research (iDiv)Halle‐Jena‐LeipzigGermany

**Keywords:** adaptation, asexual reproduction, niche shifts, plant invasion, reproductive system, species distribution modelling

## Abstract

- Biological invasions can be associated with shifts of the species’ climatic niches but the incidence of such shifts is under debate. The reproductive system might be a key factor controlling such shifts because it influences a species’ evolutionary flexibility. However, the link between reproductive systems and niche dynamics in plant invasions has been little studied so far.
- We compiled global occurrence data sets of 13 congeneric sexual and apomictic species pairs, and used principal components analysis (PCA) and kernel smoothers to compare changes in climatic niche optima, breadths and unfilling/expansion between native and alien ranges. Niche change metrics were compared between sexual and apomictic species.
- All 26 species showed changes in niche optima and/or breadth and 14 species significantly expanded their climatic niches. However, we found no effect of the reproductive system on niche dynamics. Instead, species with narrower native niches showed higher rates of niche expansion in the alien ranges.
- Our results suggest that niche shifts are frequent in plant invasions but evolutionary potential may not be of major importance for such shifts. Niche dynamics rather appear to be driven by changes of the realized niche without adaptive change of the fundamental climatic niche.

## Introduction

Biological invasions, the colonization of regions outside a species’ native range as a result of human activities, can be associated with shifts in the species’ realized climatic niches. The incidence of such niche shifts during invasions has been an issue of recent debate, with some studies suggesting prevalent niche conservatism (Petitpierre *et al*., 2012) and others documenting widespread niche change (Broennimann *et al*., 2014a; Early & Sax, 2014). The reasons for these divergent results may partly stem from methodological differences in niche modelling techniques (Guisan *et al*., 2012, 2014) but may also be associated with attributes of the species studied. A likely key trait controlling niche dynamics is the reproductive system because it influences both a species’ ability to spread into new habitats and its evolutionary flexibility (Novak & Mack, 2005; Barrett *et al*., 2008). However, the possible link between reproductive systems and the incidence and magnitude of niche dynamics in plant invasions has been little studied so far.

Flowering plants represent a broad array of reproductive systems, including sexual reproduction as well as self‐fertilization and asexual reproduction by means of clonal growth or apomixis. These different reproductive strategies might promote climatic niche changes during invasion processes via different causal pathways. On the one hand, niche dynamics may be triggered by rapid adaptive evolution of the species’ fundamental niches (Prentis *et al*., 2008). Such rapid adaptation to new niche space in the alien range is probably facilitated by high standing genetic variation and recombination, which generally are features of biparentally reproducing (outcrossing) sexual plants (Hermisson & Pennings, 2005), and which are particularly effective when multiple separate introductions allow for the hybridization of different genotypes that had been spatially separated in the native range (Novak & Mack, 2005). On the other hand, niche dynamics may rather result from changes in the realized niche following the release from biotic constraints on native distributions such as predators, pathogens or competitors, without any adaptive change to the fundamental climatic niche (Tingley *et al*., 2014). A recent analysis of European plants naturalized in North America suggested that such release from nonclimatic distribution barriers may actually be an important trigger of climatic niche shifts in alien ranges (Early & Sax, 2014). In this latter case, it is not the evolutionary flexibility but rather the extent of nonclimatic native range restrictions together with the species’ ability of rapid spatial spread in the introduced range that will promote changes in the (realized) niche.

Among reproductive systems, spread ability is commonly thought to be positively correlated to a species’ capacity to reproduce in the absence of mates or pollinators (‘Baker's law’; Baker, 1955, 1967; Bazin *et al*., 2014; Pannell *et al*., 2015) because uniparental reproduction relaxes Allee effects and allows new populations to become established from single propagules (Pannell & Dorken, 2006) which occasionally are dispersed over unusually long distances. Apomixis is a special case of uniparental reproduction where seeds are produced asexually (Barrett, 2010). Apomixis hence excludes any recombination and is a way to make clonal lineages spatially mobile. Apomictic taxa generally arise from hybridization in the first place (Asker & Jerling, 1992; Hörandl, 2006; Whitton *et al*., 2008; Hojsgaard *et al*., 2014a) and usually comprise a set of different clonal lineages (Edwards *et al*., 2006; Paun *et al*., 2006; Yu *et al*., 2014), each of which is supposedly adapted to a slightly different niche optimum (‘frozen’, ‘frozen niche variation model’; Vrijenhoek & Parker, 2009). This partitioning of niche space has been put forward as one explanation for the recurrent pattern in which apomictic taxa occupy larger and climatically more extreme native areas than their sexual counterparts (geographical parthenogenesis; Kearney, 2005; Hörandl, 2006; Hörandl *et al*., 2008). However, if niche dynamics in alien ranges are mainly mediated by rapid adaptation to new conditions, one would expect apomictic species to be particularly conservative or even to shrink their niches in the alien range because (1) only a subset of lineages will usually be introduced to the new territory and (2) recombination is suppressed and evolutionary flexibility hence low (even if many apomictic species sustain some residual sexuality; Asker & Jerling, 1992). If, in contrast, niche dynamics in alien ranges are mainly triggered by rapid spatial spread following the relaxation of nonclimatic constraints, apomixis may actually represent an advantage because it guarantees independence of mating partners during range expansion.

**Figure 1:**
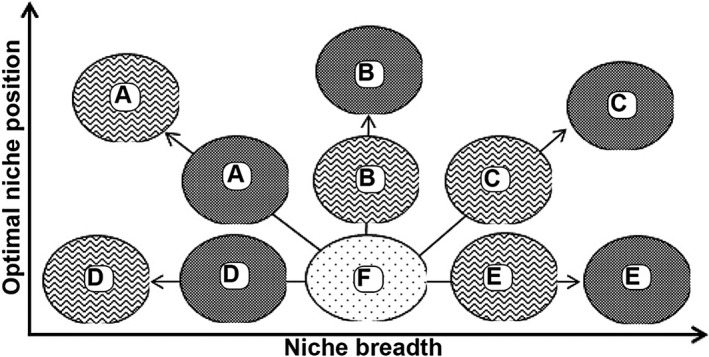
Simplified scenarios of niche change in plant invasions; the native niche (bulb with a dotted pattern) occupies a specific position and breadth (F). During an invasion, niches may remain stable (F) or changes may occur in optimal niche position (A, B and C) and/or niche breadth (A, C, D and E). Following our basic hypothesis, shifts in optimal niche position should occur both in apomictic species (bulbs with a zig‐zag stripe pattern) and in sexual species (bulbs with a checked pattern), but sexual species should show more pronounced niche broadening. Types of patterns: (A) change in position, niche contraction; unfilling of native niche space and/or expansion into a narrower, new niche space; (B) change in niche position, breadth remains the same; expansion and unfilling are balanced; (C) change in niche position, niche broadens; expansion more pronounced than unfilling; (D) niche position remains the same, niche contraction; unfilling; (E) niche position remains the same, niche broadens at margins; expansion; (F) native niche position and breadth remain the same during naturalization; stability.

The direct comparison of closely related species provides a powerful tool for investigating the influence of specific traits on niche dynamics and potential niche shifts (Funk, 2008). Here, we contrast data for 13 apomictic species and 13 closely related (congeneric or subtribal) sexual species from both tropical and temperate regions to compare their native and alien ranges based on the currently most comprehensive global data set of alien species distributions. We analyse the changes in these species’ realized niches between native and alien ranges using the ordination‐based approach of Broennimann *et al*. (2012). Given the current controversy about niche shifts in plant invasions, we first document the incidence and magnitude of niche changes among these species and classify them according to a set of different niche change scenarios (see Fig. 1 for a summary). Second, we evaluate the effect of the reproductive system on the detected niche change dynamics. With respect to this comparison, our basic hypothesis is that genetic recombination generates adaptive potential and hence allows for more pronounced changes in both niche position and niche breadth in sexual alien species. Specifically, we expect to find higher rates of expansion into novel niche space with concomitant niche broadening in species relying on sexual reproduction (Fig. 1, scenarios C and E). Following the same reasoning, we basically assume that alien apomicts will not be able to broaden their niches while their niche optima might also change because only a subset of ‘frozen’ lineages is represented in the alien range (Fig. 1, scenarios A and D). Third, and finally, we assessed if the detected niche dynamics are related to native niche breadth as an indication of the role of nonclimatic native range restrictions for these dynamics (Early & Sax, 2014).

## Materials and Methods

### Selection of study species

To investigate the relationship between niche dynamics and reproductive systems in alien plants, we selected 13 taxonomically related pairs of one sexual and one apomictic species each. We only selected pairs where both partners have documented naturalized alien distributions. Thus, prominent apomicts such as species of Taraxacum and Hieracium, where only apomictic taxa are naturalized, were excluded. In order to identify suitable study species, we screened the Apomixis Database for genera with trustworthy records (Hojsgaard *et al*., 2014b; http://www.apomixis.uni-goettingen.de), which resulted in a list of 293 genera. We then queried these genera in the Global Naturalized Alien Floras database (GloNAF; van Kleunen *et al*., 2015) to identify apomictic genera containing alien species. The GloNAF database is a newly established and comprehensive global alien plant species distribution database which contains information on the native and naturalized alien distribution in 843 nonoverlapping regions covering *c*. 83% of the terrestrial area of the world. In total, the GloNAF database contains *c*. 13 168 naturalized alien plant species following the criteria for naturalization status proposed by Richardson *et al*. (2000). By overlaying the above list of apomictic genera with the GloNAF database, we pre‐selected 41 genera, aiming at a broad sampling across the phylogeny, targeting both tropical and temperate species and large families to increase the chances of finding suitable sexual congenerics (Hojsgaard *et al*., 2014b). We then screened the literature for information on the reproductive systems of the relevant species (Supporting Information Methods S1 and Table S1). The reliability of information on reproductive systems was assessed following criteria of Hojsgaard *et al*. (2014b). To ensure comparability of the species within the pairs, we combined the selected apomicts with congeneric species or, if no other partner could be found, species belonging to the same tribe (pairs 1–3, 6 and 12). To standardize native climatic niches as far as possible, species within a pair should moreover share a native range in the same biome on the same continent (exceptions being the pairs 2, 12 and 13; see Fig. S1).

### Occurrence data and definitions of native and alien ranges

Occurrence data were downloaded for all putative species pairs from the Global Biodiversity Information Facility using the ‘rgbif’ package in R (GBIF; http://www.gbif.org, accessed February/March 2014). The complete set of synonyms was included in the download and cross‐checked with The Plant List (http://www.theplantlist.org; Table S2). Erroneous GBIF‐occurrence points (e.g. coordinates outside the country under which they had been listed) were removed using the ‘overlay’ function in the ‘sp’ package in R (R Core Team, 2014). [Correction added after online publication 28 October 2015: in the preceding sentences reference to two of the packages in R, ‘rgbif’ and ‘sp’, were updated.] The native and alien ranges of all species were defined on the basis of the regions recorded in the GloNAF database. GBIF occurrences were cross‐checked with the GloNAF database, and occurrences that could not be identified as native or alien based on GloNAF were checked by screening available literature and various databases of invasive species (Table S3). If no reliable information could be found, they were omitted. For species with few GBIF occurrences in either range, we searched for additional occurrence data in databases of the specific regions (Table S4), considering 40 occurrences in the native and the alien ranges, respectively, as the minimum number for including a species in our analysis. After these filtering procedures, 26 species (13 pairs) were kept (Table 1; see Fig. S1 for species’ ranges). When calculating the niches of these species (see Quantification of niche dynamics), we disaggregated potentially clustered occurrence points by randomly selecting one occurrence point within a radius of 0.08333° (Broennimann *et al*., 2012). The final number of occurrences used for analysis is given in Table S5.

**Table 1:** Pairing, mode of reproduction, niche change metrics and niche change scenario of the species analysed

Pair ID	Family	Species	Reproduction	*D*	Change in niche optimum	Change in niche breadth PC1	Change in niche breadth PC2	Niche change scenario	Expansion/unfilling
1	Asteraceae	*Ageratina adenophora*	Apo	0.234	**s**	c	b	**B**	**E**
1	Asteraceae	*Mikania micrantha*	Sex	0.1751	**s**	b	c	**B**	**U**
2	Asteraceae	*Chromolaena odorata*	Apo	0.3531	**ns**	b	c	**F**	
2	Asteraceae	*Eupatorium cannabinum*	Sex	0.3121	**s**	b	**b**	**C**	**E & U**
3	Asteraceae	*Erigeron annuus*	Apo	0.2199	**s**	**c**	**c**	**A**	**U**
3	Asteraceae	*Erigeron canadensis*	Sex	0.1926	**s**	**c**	**c**	**A**	**U**
4	Euphorbiaceae	*Euphorbia esula*	Apo	0.2576	**s**	**b**	**b**	**C**	**E**
4	Euphorbiaceae	*Euphorbia cyparissias*	Sex	0.4368	**s**	**b**	**b**	**C**	**E**
5	Hypericaceae	*Hypericum perforatum*	Apo	0.3459	**s**	**b**	**b**	**C**	**E**
5	Hypericaceae	*Hypericum androsaemum*	Sex	0.4275	**s**	b	**b**	**C**	
6	Melastomataceae	*Clidemia hirta*	Apo	0.1959	**s**	b	**c**	**A**	**U**
6	Melastomataceae	*Miconia calvescens*	Sex	0.1948	**ns**	**c**	**c**	**D**	**U**
7	Poaceae	*Brachiaria brizantha*	Apo	0.1398	**s**	b	b	**B**	**E & U**
7	Poaceae	*Brachiaria ruziziensis*	Sex	0.0423	**s**	**b**	b	**C**	**E & U**
8	Poaceae	*Cortaderia jubata*	Apo	0.3368	**s**	**c**	c	**A**	**U**
8	Poaceae	*Cortaderia selloana*	Sex	0.3156	**s**	**b**	**c**		**E & U**
9	Poaceae	*Eragrostis curvula*	Apo	0.5107	**s**	**b**	b	**C**	
9	Poaceae	*Eragrostis superba*	Sex	0.2379	**s**	c	b	**B**	**E & U**
10	Poaceae	*Paspalum conjugatum*	Apo	0.317	**s**	c	b	**B**	
10	Poaceae	*Paspalum urvillei*	Sex	0.3329	**s**	b	b	**B**	**E**
11	Poaceae	*Poa pratensis*	Apo	0.3649	**s**	**b**	**b**	**C**	**E**
11	Poaceae	*Poa annua*	Sex	0.3208	**s**	**b**	**b**	**C**	**E**
12	Rosaceae	*Potentilla recta*	Apo	0.3356	**s**	**c**	**b**		**U**
12	Rosaceae	*Duchesnea indica*	Sex	0.3025	**s**	c	**c**	**A**	**U**
13	Rosaceae	*Rubus pensilvanicus*	Apo	0.0026	**s**	c	b	**B**	**E & U**
13	Rosaceae	*Rubus phoenicolasius*	Sex	0.0336	**s**	**b**	**b**	**C**	**E & U**

Changes in niche optima are classified as significant (s) or nonsignificant (ns). Changes in niche breadth are summarized as contraction (c) and broadening (b), significant c/b is given in bold. Niche change scenarios follow Fig. 1; three species both broadened and contracted their niches along the two principal components analysis (PCA) axes and were not assigned to a specific niche change scenario. Niche change scenarios: A, change in optimal niche position and niche contraction; B, change in optimal niche position; C, change in optimal niche position and niche broadening; D, niche contraction; F, niche stability. Expansion (E) into novel and/or unfilling (U) of native niche space in the alien area larger than 10% is given in the last column.

Data on first introductions of the 26 selected species to all the different regions considered in our analysis (see section ‘Background climates’) were not available. However, to get an idea of whether a bias in these residence times towards either sexual or apomictic species might affect our comparison, we extracted residence times from GloNAF and several other sources (Table S6) for the subset of alien regions where they were available. From these numbers, we calculated mean residence times per species. The results demonstrate very similar mean residence times (sexual species, 91.8 yr on average; apomictic species, 100.3 yr on average; paired *t*‐test, *t *=* *0.84, *P *=* *0.41; see Table S5) and hence no indication of any relevant bias.

### Climate data preparation and variable selection

Climate data were obtained from the WorldClim data set (Hijmans *et al*., 2005, http://www.worldclim.org, accessed 2014/05/09) at 5 arcmin resolution. Additionally, global data on the annual aridity index (AI) and monthly potential evapotranspiration (PET) were downloaded from the CGIAR database (Consortium for Spatial Information, http://www.cgiar-csi.org) at 30 arcsec resolution and aggregated to 5 arcmin by taking the cell mean. An additional variable, water balance over the year (WBAL), was calculated as the sum of monthly precipitations (from the WorldClim data set) minus the monthly potential evapotranspiration (Skov & Svenning, 2004). From these variables, we selected a subset that should represent the most important climatic drivers of species’ global distributions, namely energy input, water availability, frost risk and climatic variability (bio5, maximum temperature of the warmest month; bio6, minimum temperature of the coldest month; bio7, temperature annual range; bio9, mean temperature of the driest quarter; bio15, precipitation seasonality; WBAL, annual water balance (all *r *<* *0.8, except bio6–bio7 (−0.87) and bio6–bio9 (0.94)).

### Background climates

The analysis of niche differences is based on species’ occurrence densities under certain climatic conditions standardized by the densities (= the availability) of these conditions in the native and alien ranges (see Quantification of niche dynamics). We defined the areas relevant for calculating these background densities based on the World Wildlife Fund's data set of 825 terrestrial ecoregions of the world (Olson *et al*., 2001, http://www.arcgis.com/home/item.html?id=be0f9e21de7a4a61856dad78d1c79eae), that is, the native and invasive ranges of a particular species comprised all of these regions where at least one occurrence point of the respective species is documented in our data set. We expected that such a spatially differentiated definition of background areas would reduce the possible effects of dispersal limitations on niche comparisons (Glennon *et al*., 2014). We also ran all analyses by defining whole continents as background areas (as in Petitpierre *et al*., 2012): the results differed quantitatively, but not qualitatively, with respect to our research questions. We hence only report the region‐based results for clarity.

### Quantification of niche dynamics

For characterizing the niches of our model species, we applied the approach developed by Broennimann *et al*. (2012). This method has found recent application in several studies focusing on niche shifts in alien species and niche differentiation of polyploid taxa (Petitpierre *et al*., 2012; Glennon *et al*., 2014). All analyses were performed in R 3.1.1 based on the script used by Broennimann *et al*. (2012; Methods S1).

The method works in a two‐dimensional gridded environmental space. We hence first subjected the selected climatic variables to a principal components analysis (PCA) – using the entire environmental space of both the native and alien distribution areas of a particular species (PCA‐env in the terminology of Broennimann *et al*., 2012) – and extracted the first two axes from these PCAs. The two‐dimensional PCA space was then subdivided into a 100 × 100 grid and native and alien species occurrences, respectively, in bins of this two‐dimensional environmental space were converted into densities using kernel smoothers. Species occurrence densities were subsequently scaled by densities of environmental conditions in the background area to derive a description of the (realized) niche in the native and alien ranges, respectively. This approach is particularly attractive because it accounts for differential availability of climatic conditions and differential sampling efforts in the alien and native ranges of species (compare Broennimann *et al*., 2012; Petitpierre *et al*., 2012). However, it does not alleviate a geographical bias in sampling effort, for example systematic undersampling of an area.

The overlap between the calculated native and alien niches of each species was measured using Schoener's *D* metric (Warren *et al*., 2008). This metric ranges between 0 (no overlap) and 1 (complete overlap). To statistically evaluate the calculated overlap, the highly conservative niche equivalency (random reallocation of occurrences in both ranges to evaluate whether niches are identical) and the niche similarity (random reallocation in one range and comparison of randomized niche with observed niche in other region) tests were applied (Warren *et al*., 2008; see Notes S1 and Table S7 for details).

For evaluating possible differences in the magnitude of native–alien niche changes among reproductive modes, we first bootstrapped metrics of central tendency (= optimal niche position) and variability (= niche breadth) for each species in the two ranges separately. For this purpose, we re‐sampled occurrence records in either range 100 times (with replacement) and re‐calculated PCAs each time. On each of the 100 PCAs, values of 100 random pixels, weighted by occurrence density, were taken from the environmental space. For each of the two PCA axes separately, optimal niche position in a particular range was then calculated as the median of these values and niche breadth as the difference between the 0.025 and 0.975 quantiles. To calculate the extent of niche shift, we subtracted the optimal niche position of a species in the alien range from its optimal position in the native range for each bootstrap run. A shift in optimal niche position was considered significant if the central 95% of the thus calculated differences did not include 0. To estimate niche broadening or contraction, alien niche breadth was divided by the breadth in the native range for each bootstrap run. The niche was considered significantly smaller or broader in the alien range if the central 95% of the thus calculated alien/native range ratios was < 1 or > 1, respectively.

Direct overlays of the calculated native and alien occurrence densities in the two‐dimensional PCA space allow calculation of niche unfilling and expansion. These are the proportion of the native range niche not filled in the alien range and the proportion of completely new niche space in the alien range, respectively (cf. Petitpierre *et al*., 2012; Guisan *et al*., 2014). Note that expansion into new niche space can occur without a change of total niche breadth, while changes of niche breadth necessarily imply expansion into new niche space and/or unfilling of native niche space in the alien range (Fig. 1, scenario B). We calculated such overlays, again as means from 100 bootstraps, using the ‘ecospat’ package in R (Broennimann *et al*., 2014b). Following the approach of Early & Sax (2014), we included the full climatic space of both ranges in these calculations, but an alternative analysis with analogous climates did not change the results much (cf. Notes S2 and Table S8). Fig. 1 provides an overview of the different scenarios of niche dynamics that were evaluated.

### Effects of reproductive systems and native niche breadth on niche dynamics

We used linear mixed‐effects models (LMMs) to test for significant differences in the metrics of niche dynamics among sexual and apomictic species. To assess differences in niche overlap, Schoener's *D* values for apomictic species were subtracted from the *D* values for congeneric sexual species. To assess differences in niche shifts, mean bootstrapped position shifts (native minus alien optimal position on each PCA axis) and mean bootstrapped changes in niche breadth (invasive/native niche breadth) of apomictic species were subtracted from the mean bootstrapped position and breadth shifts of the congeneric sexual species. We then applied LMMs to assess whether the means of these differences in niche change metrics between congeneric species pairs deviated significantly from zero. We accounted for phylogenetic relatedness by including taxonomic family as a grouping factor in the model. To compare differences in niche expansion and unfilling in relation to reproductive mode and native niche breadth, LMMs were run with reproductive mode or niche breadth as a fixed‐effects predictor and pair ID as a random effects grouping factor.

## Results

### Schoener's *D*, equivalency and similarity of native and alien niches

In general, native and alien niches did not show strong overlap in our study species (Schoener's *D* < 0.5 except for *Eragrostis curvula*:* D* = 0.51; Table 1) For all species, the restrictive equivalency test was significant, rejecting the hypothesis that niches are equivalent in the native and alien ranges. Nevertheless, according to the niche similarity test, niches were still more similar than expected by chance in 18 of the 26 species (Notes S1; Table S7). Importantly, there was no difference in the niche overlap metric *D* between sexual and apomictic species (*t*‐value −0.78; df = 11; *P *=* *0.45).

### Niche change patterns, expansion and unfilling

For the 26 study species, we could observe five of the six scenarios illustrated in Fig. 1: six species significantly contracted their niches, 10 significantly broadened their niches, two species both significantly broadened and contracted their niches on one or the other PCA axis (Fig. 2.5), and for eight species, niche breadth remained the same. Moreover, all but two species showed significant changes of optimal niche position along at least one of the two PCA axes (Fig. 1, scenarios A, B and C; Table 1). Only one species significantly contracted its niche without a significant change in niche position (scenario D, Fig. 2.4) and one species remained static (scenario F). None of the species significantly broadened its niche without a change of niche position (scenario E).

**Figure 2:**
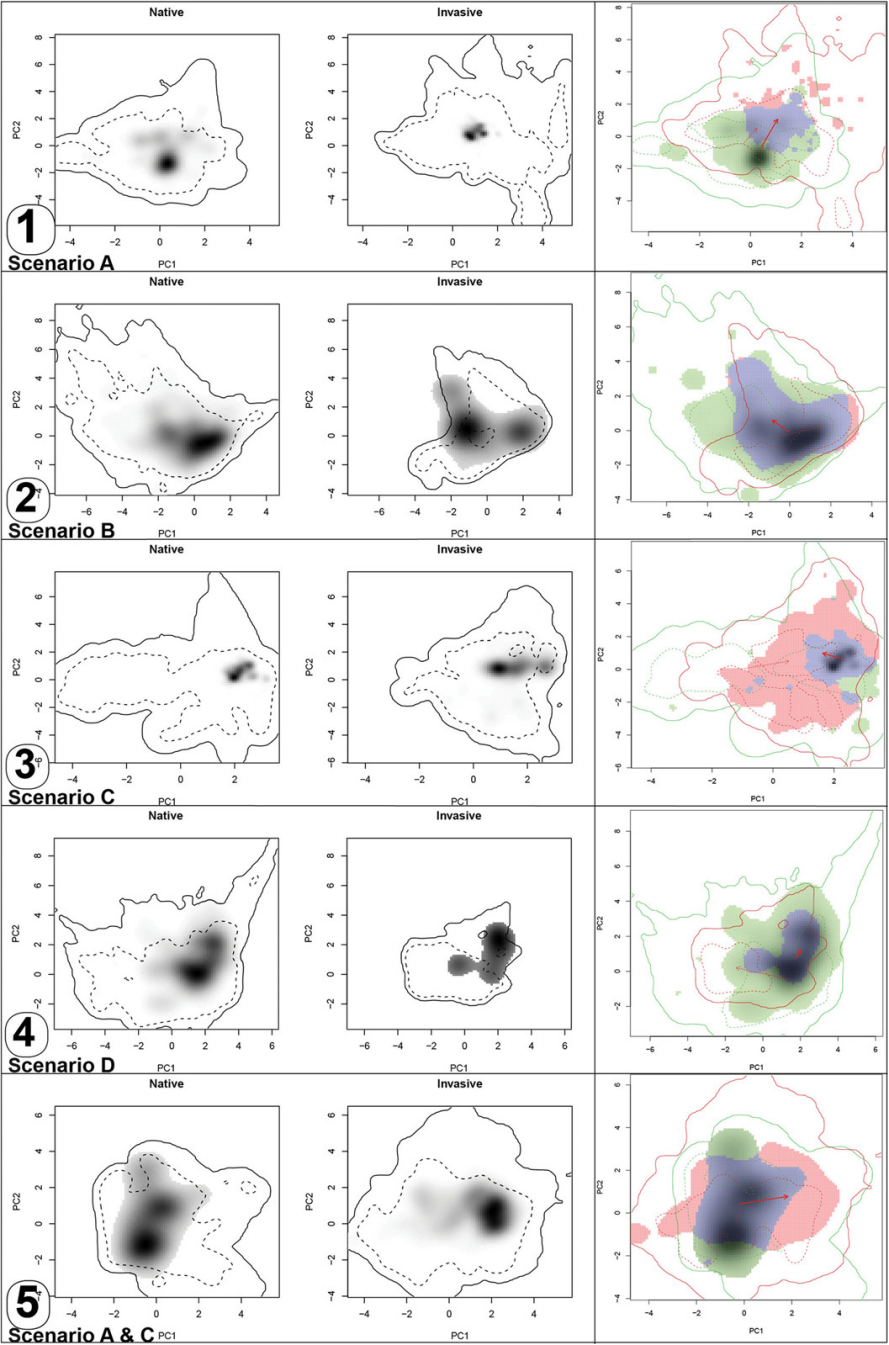
Five selected scenarios of niche change observed in the study species and associated expansion (red), stability (blue) and unfilling (green) in the alien niche. Darker shading indicates higher density of occurrences of the species; solid contour lines, 100% of available environment; dashed contour lines, 50% of most common background environment. Arrows link the centroids (optimal niche position) of the native and alien distributions (continuous arrow) and link native and alien extents (dashed arrow). (1) Scenario A: *Erigeron annuus*, change of niche position, niche contraction and unfilling in the alien niche. (2) Scenario B: *Mikania micrantha*, change in niche optimum during invasion but no significant alteration of niche breadth, niche unfilling. (3) Scenario C: *Euphorbia esula*, significant change in niche position and broadening, expansion into novel niche space. (4) Scenario D: *Miconia calvescens*, niche optima did not change but niches significantly contracted during invasion, leading to unfilling. (5) Scenarios A and C: *Cortaderia selloana*, significant change in niche optimum and both niche broadening (PC1) and niche contraction (PC2) occurred.

Seven species expanded their native climatic niche by > 10% in the alien range and eight species unfilled > 10% of the native niche space in the alien range. Additionally, seven species both expanded their native niches by > 10% and unfilled > 10% of their native niche space (Table 1).

### Effects of reproductive systems and native niche breadth on niche dynamics

**Figure 3:**
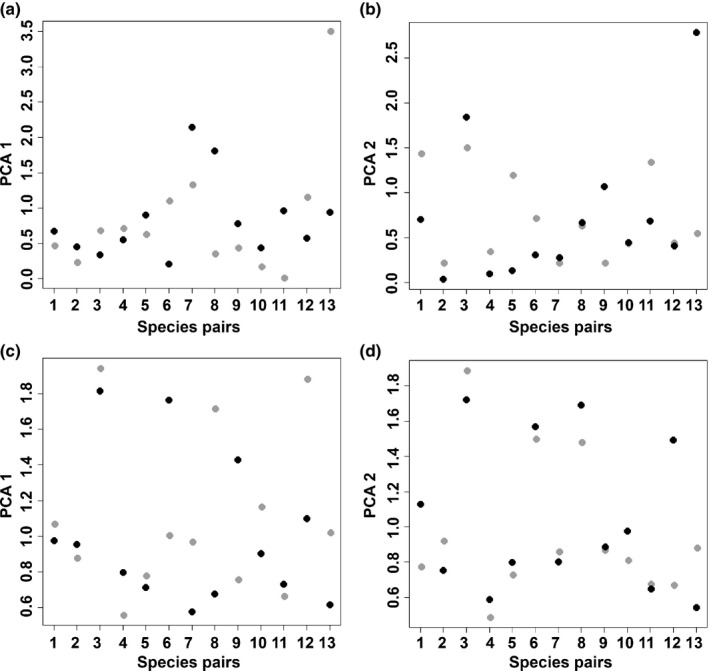
Pairwise relations of changes of optimal niche position (a, b) and niche breadth (c, d) during invasion. For changes in niche optima, a value close to 0 indicates little change in niche optimum between native and alien ranges. Concerning niche breadth, a value > 1 indicates broadening of the niche during invasion and a value smaller than 1 indicates contraction. The first two axes of a principal components analysis (PCA) are shown; grey, apomictic species; black, sexual species. Numbers refer to the pair ID (Table 1).

Of the 10 species that significantly broadened their niches (while changing niche optima: scenario C), four are apomictic, and of the five species that significantly contracted their niches (while changing niche optima: scenario A), three are apomictic (Table 1). The change in niche optimum and niche breadth between the native and alien ranges of each apomictic/sexual pair is shown in Fig. 3. We did not find a significant effect of reproductive mode either on changes in niche optima (PCA 1: *t*‐value −0.59; df = 11; *P = *0.56; PCA2: *t*‐value 0.076; df = 11; *P = *0.94) or on changes in niche breadth (PCA1: *t*‐value 0.869; df = 11; *P = *0.40; PCA2: *t*‐value −0.69; df = 11; *P = *0.50).

**Figure 4:**
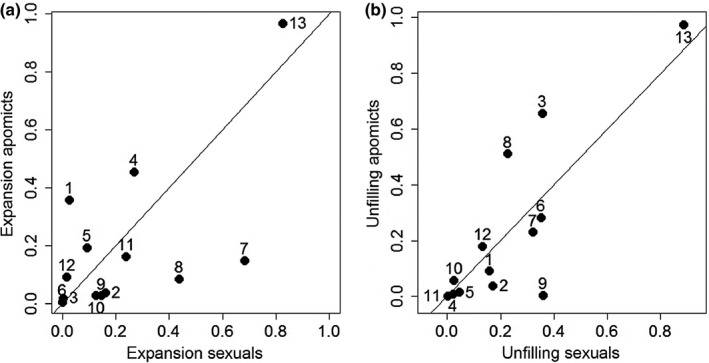
Relative expansion and unfilling for sexual (*x*‐axis) and apomictic (*y*‐axis) species. Values close to the 45° line indicate similar niche dynamics in the sexual and apomictic species of a particular congeneric (or contribal) pair, while values closer to the *y*‐axis indicate stronger niche change in the apomictic species, and values closer to the *x*‐axis indicate stronger niche change in the sexual species. Numbers refer to the pair ID in Table 1.

Patterns of niche expansion and unfilling are shown in Fig. 4. Of the seven species that showed > 10% expansion of niche space, four were apomictic, and of the eight species unfilling > 10%, four were apomictic (Table 1). Of the seven species that both expanded and unfilled > 10% of their niches, two were apomictic. LMMs for the comparison of niche expansion and niche unfilling patterns between sexual and apomictic species revealed no significant difference in any of these metrics (expansion: *t*‐value 0.54; df = 24; *P = *0.59; unfilling: *t*‐value 0.015; df = 24; *P = *0.98).

By contrast, we found that species with smaller native niches expanded their niches significantly more in the alien range (*t*‐value −3.248; df = 24; *P *<* *0.01) while there was no significant effect on niche unfilling (*t*‐value 0.429; df = 24; *P = *0.67).

## Discussion

The patterns of niche dynamics documented here suggest that shifts in climatic niches are relatively frequent among plant invaders. This finding is in line with the results of a recent study detecting pronounced niche shifts in European species invading North America (Early & Sax, 2014). By contrast, Petitpierre *et al*. (2012) found much higher levels of niche conservatism in a study on Holarctic plant invaders. This difference may partly be explained by the fact that Petitpierre *et al*. (2012) limited their comparison to niche changes within analogous climates, that is, to climatic conditions that are realized in both the native and alien ranges, while both in our study and that of Early & Sax (2014) the full climatic space of the alien range was included in the analysis. Whereas the former approach is more directly related to the issue of niche change, the latter addresses the question of whether or not climatic alien ranges can be predicted from native distributions given that available climates may differ among alien and native ranges. However, a supplementary analysis restricting comparisons to analogous climates did not change the results of our study qualitatively (Notes S2). We hence conclude that the detected niche dynamics are not primarily attributable to the colonization of climatic space not available in the native range but actually represent changes of realized niches.

Among the scenarios of niche dynamics considered (Fig. 1), a sizable majority of species (17) underwent both shifts in niche optimum and changes in niche breadth (scenarios A and C). These results strongly support the argument for considering both niche optimum and niche breadth when drawing inferences from such types of analyses (Glennon *et al*., 2014). More importantly, in our context, we could not detect any indication of the hypothesized difference between sexual and apomictic species with respect to these frequent changes in niche breadth: niche broadening (scenarios C and E) and niche restriction (scenarios A and D) seem approximately equally distributed among the two types of reproductive systems. If our assumption of a higher evolutionary potential of sexual species holds, this result suggests that, in contrast to our basic assumption, adaptive evolution is not the predominant driver of the detected niche dynamics. A more direct rejection of this hypothesis would require correlating measurements of genetic diversity (as an indicator of adaptive potential) with the calculated metrics of niche dynamics. While available data do not allow such an evaluation, some documented patterns can nevertheless be discussed. High genetic variation in Chinese populations of sexual *Mikana micrantha* Kunth (Wang *et al*., 2008), for example, does not relate to niche broadening but to contraction and niche unfilling in our study. Similarly, events of residual sexual reproduction in the alien range have given rise to a certain amount of genetic diversity and novel phenotypes in apomictic *Erigeron annuus* (L.) Pers. (Edwards *et al*., 2006); however, the species underwent significant niche unfilling. Despite losses of genetic diversity in the apomict *Ageratina adenophora* (Spreng.) R.M. King & H. Rob. in China (Zhao *et al*., 2013), we found an exceptionally high proportion of niche expansion. A strong loss of genetic diversity in the invasive range also occurred in *Chromolaena odorata* (L.) R.M.King & H.Rob. (Yu *et al*., 2014), but our data suggest that niche breadth remained the same. By contrast, North American populations of apomictic *Hypericum perforatum* L. are genetically relatively diverse (Molins *et al*., 2014) and the species has undergone significant alterations of its niche optimum and has broadened its niche with moderate expansion into new niche space. In accordance with our documentation of niche changes in both sexual and apomictic species, these patterns suggest that there might be some connection between genetic diversity and niche dynamics in individual cases but that this connection is not straightforward and that rapid evolutionary adaptation is hence unlikely to represent the main driver of niche dynamics in the species studied here.

The absence of any difference in niche expansion rates among sexual and apomictic species is, however, compatible with the hypothesis of Early & Sax (2014) that release from nonclimatic restrictions allows species to realize their already existing climatic potential more completely in the alien than in the native range. This interpretation is further underpinned by the finding that, as in Early & Sax (2014), species with narrower climatic niches in the native range showed significantly higher rates of niche expansion in the alien range. Nevertheless, we could not find support for the hypothesis that apomicts may expand their realized niches even more markedly as a result of the greater spread capacity of uniparentally reproducing species (Baker, 1967; Bazin *et al*., 2014). We suppose, however, that this result is not inconsistent with the possible advantages of uniparental reproduction for niche expansion rates, because some of our sexual invaders are self‐compatible (Table S1) and will hence similarly benefit from independence of mating partners. In addition, the opposing advantages of higher evolutionary flexibility of sexually reproducing species and higher spread rates of apomictic species may balance each other to a certain degree and additionally mask differences in niche dynamics such that they become undetectable with the rather small sample size of this study. Finally, the advantage of higher spread rates of uniparentally reproducing species might theoretically be masked by a bias of shorter residence times on the part of these species, but our data do not suggest such a bias (Table S5).

While release from nonclimatic barriers may explain niche expansions in the alien range, it does not help in understanding cases of niche contraction in our data. Short residence times are an obvious candidate for explaining apparent niche contractions, because species may simply not have had enough time to fill their potential niches (Williamson *et al*., 2009). Indeed, pronounced niche unfilling in species such as *Mikania micrantha* (mean residence time 18 yr; Table S5) might be explained as such a transient phenomenon. However, unfilling also occurs in species with much longer residence times such as *Potentilla recta* L. (mean residence time 131 yr; Table S5), indicating that factors other than short residence times can additionally trigger niche contractions. Among these, the introduction of only a subset of the ‘frozen’ clonal lineages is a likely explanation in the case of apomicts (such as *Potentilla recta*): as these lineages partition the species’ niche space (Vrijenhoek & Parker, 2009), any subset will only represent part of the overall species’ niche. In the case of outcrossing sexual species, lack of pollinators and mates can potentially induce extended lag‐phases (which delay the species’ spread and hence the unfilling of its climatic niche in the alien range by decades or even longer; Burns *et al*., 2011; Pyšek *et al*., 2011; Bufford & Daehler, 2014).

An important caveat to all niche‐shift analyses that are based on occurrence data is the quality of these data. Although we have used various data sources and cross‐checked all occurrences with the GloNAF database (van Kleunen *et al*., 2015), GBIF, which has often been criticized for being incomplete and erroneous, contributed the vast majority of records to our analysis. In particular, GBIF has a low coverage in parts of Asia and Africa. Nevertheless, we do not think that errors and biases implicit to this database have qualitatively affected our comparison between sexual and apomictic species because: there was no indication of a systematic bias in sampling towards either the native or the alien ranges (Fig. S1; Table S5); including continent of origin in the analysis demonstrated some significant differences, but none of them is clearly linked to a known bias in GBIF (Notes S3); notoriously undersampled Russia is part of native and alien ranges, respectively, of an approximately equal number of sexual and apomictic species (native to Russia: the sexual species *Eupatorium cannabinum* L. and *Poa annua* L. and the apomictic species *Euphorbia esula* L. and *Hypericum perforatum* L.; alien to Russia: the sexual species *Duchesnea indica* (Andrews) Th.Wolf and *Erigeron canadensis* (L.) Cronquist and the apomictic species *Erigeron annuus*). Finally, there was no evidence for systematic undersampling of species with small native ranges which, theoretically, could have produced the detected inverse correlation of native niche breadth and magnitude of niche dynamics.

### Conclusions

While our results suggest that niche shifts are frequent in plant invasions, they do not support our basic hypothesis that sexual species, which probably have higher evolutionary potential, will be characterized by a higher incidence and magnitude of such shifts. Instead, they are at least partly in line with the hypothesis that niche dynamics, and in particular niche expansion, often occur in species that are restricted by nonclimatic barriers in their native ranges and that these dynamics hence do not necessarily involve any evolutionary response to the novel environments but simply represent different realizations of the same fundamental niche. This does not imply that evolutionary adaptations are generally irrelevant for alien niche shifts (Prentis *et al*., 2008), but indicate that they may not be the dominant process behind observed niche dynamics. Nevertheless, to fully understand the drivers of niche shifts in any particular plant invasion, historical, evolutionary and ecological drivers as well as the origin and properties of the target species and the influence of biotic interactions should ideally be considered in concert (Broennimann *et al*., 2014a). The complexity of such interacting drivers might create ‘noise’ that could easily mask more subtle effects of single factors such as the reproductive system.

## Author contributions

S.D., A.S.D., F.E. and E.H. designed the research. The apomixis database was constructed by E.H., D.H. and S.K., and the GloNAF database by M.v.K., P.P., W.D., F.E., M.W., E.W. and J.P. The modelling and data analyses were performed by A.S.D. A.S.D. and S.D. led the writing of the paper, and J.P. and B.K. contributed to the writing of the paper. All authors commented on and contributed to earlier versions of the manuscript.

## Supporting information

Please note: Wiley Blackwell are not responsible for the content or functionality of any supporting information supplied by the authors. Any queries (other than missing material) should be directed to the *New Phytologist* Central Office.

**Fig. S1** Maps of the native and alien ranges of the 26 study species based on GloNAF.
**Table S1** Species pairs, phylogenetic relationships, details of the reproductive system and continent of origin
**Table S2** Results of synonymy checks with ThePlantList
**Table S3** Additional sources for range definitions of the 26 species
**Table S4** Additional occurrence data for nine species
**Table S5** Number of occurrences and mean residence times of the 26 species
**Table S6** Additional sources for residence time calculations
**Table S7** Results from the niche equivalency and the two niche similarity tests
**Table S8** Proportion of expansion and unfilling calculated for analogous and full climate spaces, respectively
**Methods S1** Additional information on the compilation of the 26 species’ data sets, literature and database checks and modelling procedure.
**Notes S1** Tests of equivalency and similarity of native and alien niches.
**Notes S2** Results of test on expansion/unfilling using analogous or full climatic spaces, respectively.
**Notes S3** Effects of continent of origin on niche dynamics.
