# Supplementary material for: Niche dynamics of alien species do not differ among sexual and apomictic flowering plants
Source: New Phytol. 2015 Oct 28;209(3):1313–23. doi: 10.1111/nph.13694 (PMC4950116; doi:10.1111/nph.13694)
Supplement: Supplementary file 1 — Fig. S1 Maps of the native and alien ranges of the 26 study species based on GloNAF. Table S1 Species pairs, phylogenetic relationships, details of the reproductive system and continent of origin Table S2 Results of synonymy checks with ThePlantList Table S3 Additional sources for range definitions of the 26 species Table S4 Additional occurrence data for nine species Table S5 Number of occurrences and mean residence times of the 26 species Table S6 Additional sources for residence time calculations Table S7 Results from the niche equivalency and the two niche similarity tests Table S8 Proportion of expansion and unfilling calculated for analogous and full climate spaces, respectively Methods S1 Additional information on the compilation of the 26 species’ data sets, literature and database checks and modelling procedure. Notes S1 Tests of equivalency and similarity of native and alien niches. Notes S2 Results of test on expansion/unfilling using analogous or full climatic spaces, respectively. Notes S3 Effects of continent of origin on niche dynamics. [file NPH-209-1313-s001.pdf]

New Phytologist

New Phytologist **Supporting Information**

Niche dynamics of alien species do not differ among sexual and apomictic flowering plants

Agnes S. Dellinger, Franz Essl, Diego Hojsgaard, Bernhard Kirchheimer, Simone Klatt, Wayne Dawson, Jan Pergl, Petr Pyšek, Mark van Kleunen, Ewald Weber, Marten Winter, Elvira Hörandl and Stefan Dullinger

Article acceptance date: 31 August 2015

The following Supporting Information is available for this article:

Methods S1 Additional information on the compilation of the 26 species' datasets, literature and database checks and modelling procedure.

Table S1 Species pairs, phylogenetic relationship, details on the reproductive system and continent of origin.

Table S2 Results of synonymy checks with ThePlantList.

Table S3 Additional sources for range definitions of the 26 species.

Table S4 Additional occurrence data for 9 species.

Table S5 Number of occurrences and mean residence times of the 26 species.

Table S6 Additional sources for residence time calculations.

Fig. S1 Maps of the native and alien ranges of the 26 study species based on GloNAF.

Notes S1 Tests of equivalency and similarity of native and alien niches.

Table S7 Results from the niche equivalency and the two niche similarity tests.

Notes S2 Results of test on expansion/unfilling using analogous or full climatic spaces, respectively.

Table S8 Proportion of Expansion and Unfilling calculated for analogous and full climate spaces, respectively.

Notes S3 Effects of continent of origin on niche dynamics.

Methods S1

Compilation of the GloNAF dataset

The GloNAF database is a newly established and comprehensive global alien plant species distribution database which contains information on the native and naturalized alien distribution in 843 non-overlapping regions covering ca 83% of the terrestrial area of the world. In total, the GloNAF database contains *c*. 13,168 naturalized alien plant species following the criteria on naturalization status proposed by Richardson *et al*. (2000). We extracted alien plant species numbers and species identities from national checklists, standard floras, online databases, publications, reports (white and grey literature) and by contacting regional experts (see van Kleunen *et al.*, 2015). Alien plant species lists for different regions often follow diverging taxonomic concepts and use different species names. Therefore, synonymization of the large number of alien plant species of the data sources to be included in GloNAF was a major challenge. We performed species synonymization using the R package Taxonstand (http://cran.r-project.org/web/packages/Taxonstand/index.html, Cayuela & Oksanen, 2014), which follows The Plant List (The Plant List, www.theplantlist.org/), the most comprehensive list of known and accepted plant species names.

Screening databases for species pairs

The overlay of the GloNAF and the Apomixis database resulted in 1980 alien species belonging to 190 genera from which one or more apomictic species are known. Of these, 44 genera were readily excluded as they only contained one alien species. From the remaining 146 genera, we pre-selected 41 genera aiming at a broad sampling across the phylogeny, targeting both tropical and temperate species and large families to raise chances of finding suitable partners (Hojsgaard *et al*., 2014). We then screened the literature for information on the reproductive system of the relevant species. Literature was obtained by queries in ISI Web of Science (“genus” AND apomi*, “species name” AND apomi*, “genus” AND reproduc*, “species name” AND reproduc*) and search in google scholar using the same search terms. Relevant references found in the literature, including non-digital monographs, were also consulted.

Occurrence data preparation

Using The Plant List, only occurrences of valid synonyms were kept (see Table S1). Erroneous occurrence points (e.g. in the ocean or outside the country they are coded with) were removed using the overlay function in the ‘rgbif’ package in R.

Modelling procedures

Equivalency test

The niche equivalency test explores if the niches in the native and alien range of a species are identical by randomly reallocating the occurrences in both ranges. This procedure is repeated 100 times and the recalculated niche overlap values (D) are compared with the observed D. If the observed D is lower than the randomized D in 95% of the cases, the niches are assumed to be not identical (= equivalent), although they might still be similar.

Similarity test

The less conservative niche similarity test randomly reallocates occurrences in only one range, re-calculates species densities and hence the niche in this range. The overlap between this randomized niche and the observed niche in the second region is compared with the overlap calculated based on the observations in both regions. Like in the equivalency test, randomization is done 100 times for both the native (random reallocation of the native densities) and alien (random reallocation of the alien densities) range. If the observed D is larger than the randomized one in 95%, the alien and native niches are more similar to one another than expected by chance. As both ranges are randomized separately, this test yields two results.

References

Cayuela L, Oksanen J. 2014. Taxonstand: Taxonomic Standardization of Plant Species Names. [WWW document] URL https://cran.r-project.org/web/packages/Taxonstand/index.html [accessed March 2014].

Hojsgaard D, Klatt S, Baier R, Carman JG, Hörandl E. 2014. Taxonomy and biogeography of apomixis in angiosperms and associated biodiversity characteristics. *Critical Reviews in Plant Sciences* **33:** 414–427.

Richardson DM, Pyšek P, Rejmánek M, Barbour MG, Panetta FD, West CJ. 2000. Naturalization and invasion of alien plants: concepts and definitions. *Diversity and Distribution* **6**: 93–107.

The Plant List. 2013. Version 1.1. [WWW document] URL http://www.theplantlist.org/ [accessed March 2014].

van Kleunen M, Dawson W, Essl F, Pergl J, Winter M, Weber E, Kreft H, Weigelt P, Kartesz J, Nishino M et al. 2015. The global naturalized flora: accumulation and flows of alien plants around the world. *Nature*. doi: 10.1038/nature14910.

Sources for determining the reproductive system of the study species

Pair 1

Mikania micrantha

Hong L, Shen H, Ye W, Cao H, Wang Z. 2007. Self-incompatibility in *Mikania micrantha* in South China. *Weed Research* **47**: 280–283.

Wang T, Chen G, Zan Q, Wang C, Su Y-J, Filatov DA. 2012. AFLP genome scan to detect genetic structure and candidate loci under selection for local sdaptation of the invasive weed *Mikania micrantha*. *PLoS ONE* **7**: e41310.

Ageratina adenophora

Holmgren I. 1919. Zytologische Studien über die Fortpflanzung bei den Gattungen *Erigeron* und *Eupatorium*. Svenska vetenskapsakademiens handlingar 59: 7.

Noyes RD. 2007. Apomixis in the Asteraceae: diamonds in the rough. *Functional Plant Science and Biotechnology***1**: 207–222.

Huang WK, Wan FH, Guo JY, Gao BD, Xie BY, Peng DL. 2009. AFLP analyses of genetic variation of *Eupatorium adenophorum* (Asteraceae) populations in China. Canadian Journal of Plant Sciences **89**: 119-126.

Pair 2

Eupatorium cannabinum

http://www.nrcresearchpress.com/doi/abs/10.1139/b76-313#.UxR2EYX-anw. [accessed March 2014]

Chromolaena odorata

Noyes RD. 2007. Apomixis in the Asteraceae: diamonds in the rough. *Functional Plant Science and Biotechnology* **1**: 207–222.

http://www.nrcresearchpress.com/doi/abs/10.1139/b75-072#.UxSUAYX-anx. [accessed March 2014]

Yu X, He T, Zhao J, Li Q. 2014. Invasion genetics of *Chromolaena odorata* (Asteraceae): extremely low diversity across Asia. *Biological Invasions* **16**: 2351–2366.

Pair 3

Erigeron annuus

Noyes RD. 2007. Apomixis in the Asteraceae: diamonds in the rough. *Functional Plant Science and Biotechnology* **1**: 207–222.

Conyza canadensis

Yurukova-Grancharova P, Yankova-Tsvetrkova E, Vladimirov V. 2013. Reproductive features of three invasive alien species of Erigeron (Asteraceae) in Bulgaria. *Comptes rendus de l'Académie bulgare des Sciences* **66**: 203–210.

Regehr D, Bazzaz F. 1979. The population dynamics of *Erigeron canadensis*, a successional winter annual. *Journal of Ecology* **67**: 923–933.

Pair 4

Euphorbia cyparissias

Meunscher WC. 1936. The production of seed by *Euphorbia cyparissias*. *Rhodora* **38**: 161-163.

Ehrenfeld J. 1976. Reproductive biology of three species of *Euphorbia* subgenus *Chamaesyce* (Euphorbiaceae). *American Journal of Botany* **63**: 406–413.

Euphorbia esula

Carmichael J, Selbo S. 1999. Ovule, embryo sac, embryo, and endosperm development in leafy spurge (*Euphorbia esula*). *Canadian Journal of Botany* **77**: 599–610.

Selbo S, Carmichael J. 1999. Reproductive biology of leafy spurge (*Euphorbia esula* L.): breeding system 1 (Euphorb analysis. *Canadian Journal of Botany* **77**: 1684–1688.

Pair 5

Hypericum perforatum

Matzk F, Hammer K, Schubert I. 2003. Coevolution of apomixis and genome size within the genus *Hypericum*. *Sexual Plant Reproduction* **16**: 51–58.

Scheriau C. 2011. *Evolutionary history of* Hypericum perforatum. PhD thesis, University of Heidelberg, Heidelberg, Germany.

Hypericum androsaemum

Olsen R, Ranney T. 2006. Fertility and inheritance of variegated and purple foliage across a polyploid series in *Hypericum androsaemum* L. *Journal of the American Society of Horticultural Sciences* **131**: 725–730.

Matzk F, Hammer K, Schubert I. 2003. Coevolution of apomixis and genome size within the genus *Hypericum*. *Sexual Plant Reproduction* **16**: 51–58.

Trueblood CE, Ranney T, Lynch NP. 2010. Evaluating fertility of triploid clones of *Hypericum androsaemum* L. for use as non-invasive landscape plants. *Horicultural Sciencies* **45**: 1026–1028.

Pair 6

Miconia calvescens

Meyer J-Y. 1998. Observations on the reproductive biology of *Miconia calvescens* DC (Melastomataceae), an alien invasive tree on the Island of Tahiti (South Pacific Ocean). *Biotropica* **30**: 609–624.

Clidemia hirta

Renner SS. 1989. A survey of reproductive biology in Neotropical Melastomataceae and Memecyclaceae. *Annals of the Missouri Botanical Garden* **76**: 496–518.

DeWalt SJ. 2006. Population dynamics and potential for biological control of an exotic invasive shrub in Hawaiian rainforests. *Biological Invasions* **8**: 1145–1158.

Pair 7

Brachiaria brizantha

Brown WV, Emery WHP. 1958. Apomixis in the Gramineae: Panicoideae. *American Journal of Botany* **45**: 253–263.

Brachiaria ruziziensis

Schank SC, Sotomayor-Rios A. 1968. Cytological studies on *Brachiaria* species. *Soil Crop Science Society of Florida Proceedings* **28**: 156–162.

Ferguson JE, Crowder LV. 1974. Cytology and breeding behaviour of *Brachiaria ruziziensis* Germain et Evrard. *Crop Science* **14**: 893–895.

Pair 8

Cortaderia jubata

Okada M, Lyle M, Jasieniuk M. 2009. Inferring the introduction history of the invasive apomictic grass *Cortaderia jubata* using microsatellite markers. *Diversity and Distributions* **15**: 148–157.

Connor HE. 1973. Breeding Systems in *Cortaderia* (Gramineae). *Evolution* **27**: 663–678.

Cortaderia selloana

Lambrinos J. 2001. The expansion history of a sexual and asexual species of *Cortaderia* in California, USA. *Journal of Ecology* **89**: 88–98.

Connor HE. 1973. Breeding Systems in *Cortaderia* (Gramineae). *Evolution* **27**: 663–678.

Pair 9

Eragrostis superba

Streetman LJ. 1970. Cytogenetics of *Eragrostis*. In: Dalrymple RL, ed. *Proceedings of the First Weeping Lovegrass Symposium*. Oklahoma, USA: The Samuel Roberts Noble Foundation, 10–13.

Eragrostis curvula

Voigt PW, Bashaw EC. 1972. Apomixis and sexuality in *Eragrosis curvula*. *Crop Science* **12**: 843–847.

http://www.tropicalforages.info/key/Forages/Media/Html/Eragrostis_curvula.htm. [accessed 20 March 2014].

Pair 10

Paspalum urvillei

Ortiz JPA, Quarin CL, Pessino SC, Acuna C, Martinez EJ, Espinoza F, Hojsgaard DH, Sartor ME, Caceres ME, Pupilli F. 2013. Harnessing apomictic reproduction in grasses: what we have learned from *Paspalum*. *Annals of Botany* **112**: 767–787.

Paspalum conjugatum

Ortiz JPA, Quarin CL, Pessino SC, Acuna C, Martinez EJ, Espinoza F, Hojsgaard DH, Sartor ME, Caceres ME, Pupilli F. 2013. Harnessing apomictic reproduction in grasses: what we have learned from *Paspalum*. *Annals of Botany* **112**: 767–787.

Pair 11

Poa annua

Warwick SI. 1979. The biology of Canadian weeds. 37. *Poa annua* L. *Canadian Journal of Plant Sciences* **59**: 1053–1066.

Ellis WM. 1973. The breeding system and variation in populations of *Poa annua* L. *Evolution* **27**: 656–662.

Poa pratensis

Porceddu A, Albertini E, Barcaccia G, Falistocco E, Falcinelli M. 2002. Linkage mapping in apomictic and sexual Kentucky bluegrass (*Poa pratensis* L.) genotypes using a two way pseudo-testcross strategy based on AFLP and SAMPL markers. *Theoretical and Applied Genet*ics **104**: 273–280.

Pair 12

Duchesnea indica

Naruhashi N, Sugimoto M. 1996. The floral biology of *Duchesnea* (Rosaceae). *Plant Species Biology* **11**: 173–184.

pers. com. Christoph Dobeš

Potentilla recta

Christoff M, Papasova G. 1943. Die genetischen Grundlagen der apomiktischen Fortpflanzung in der Gattung *Potentilla*. *Zeitschrift für Induktive Abstammungs- und Vererbungslehre* **81**: 1–27.

Popoff A. 1935. Über die Fortpflanzungsverhältnisse der Gattung *Potentilla*. *Planta* **24**: 510–522.

pers. com. Christoph Dobeš

Pair 13

Rubus phoenicolasius

Wint AA. 2008. *Genetic diversity in native and invasive* Rubus *(Rosaceae).* Master Thesis. Western Kentucky University, Bowling Green, Kentucky, USA.

Rubus pensilvanicus

Clark LV, Jasieniuk M. 2012. Spontaneous hybrids between native and exotic *Rubus* in the Western United States produce offspring both by apomixis and by sexual recombination. *Heredity* **109**: 320–328.

Table S1 Species pairs, phylogenetic relationship, details on reproductive system and continent of origin.

Pair nr	species	Order	Family	Type of apomixis	Self-compatibility	Continent of origin	Phylogenetic relationship
1	*Ageratina adenophora*	**Asterales**	Asteraceae	diplospory, autonomous; facultative?	SC	North America	sister genus
1	*Mikania micrantha*	**Asterales**	Asteraceae	-	SIC	South America	sister genus
2	*Chromolaena odorata*	**Asterales**	Asteraceae	diplospory, autonomous		South America	congeneric
2	*Eupatorium cannabinum*	**Asterales**	Asteraceae	-	SIC?	Eurasia-Africa	congeneric
3	*Erigeron annuus*	**Asterales**	Asteraceae	obligate diplospory, autonomous	SC	North America	congeneric
3	*Conyza canadensis*	**Asterales**	Asteraceae	-	SC	North America	congeneric
4	*Euphorbia esula*	**Malpighiales**	Euphorbiaceae	pseudogamy	SC	Eurasia	congeneric
4	*Euphorbia cyparissias*	**Malpighiales**	Euphorbiaceae	-	SC	Europe	congeneric
5	*Hypericum perforatum*	**Malpighiales**	Hypericaceae	apospory; pseudogamy; facultative	SC	Eurasia-Africa	congeneric
5	*Hypericum androsaemum*	**Malpighiales**	Hypericaceae	-	SIC	Europe	congeneric
6	*Clidemia hirta*	**Myrtales**	Melastomataceae	adventitious embryony; autonomous; probably facultative	not known	South America	contribal
6	*Miconia calvescens*	**Myrtales**	Melastomataceae	-	SIC	South America	contribal
7	*Brachiaria brizantha*	Poales	Poaceae	apospory; facultative	SC	Africa	congeneric
7	*Brachiaria ruziziensis*	Poales	Poaceae	-	SIC	Africa	congeneric
8	*Cortaderia jubata*	Poales	Poaceae	obligate(?) apospory		South America	congeneric
8	*Cortaderia selloana*	Poales	Poaceae	-	SIC	South America	congeneric
9	*Eragrostis curvula*	Poales	Poaceae	diplospory; both obligate and facultatively apomictic individuals; also sexual		Africa	congeneric
9	*Eragrostis superba*	Poales	Poaceae		SIC?	Africa	congeneric
10	*Paspalum conjugatum*	Poales	Poaceae	diplospory; facultative		South America	congeneric

10	*Paspalum urvillei*	Poales	Poaceae		SC	South America	congeneric
11	*Poa pratensis*	Poales	Poaceae	apospory; pseudogamy; facultative	SC	Eurasia-Africa	congeneric
11	*Poa annua*	Poales	Poaceae	-	SC	Eurasia-Africa	congeneric
12	*Potentilla recta*	**Rosales**	Rosaceae	pseudogamy; facultative; both diplo- and apospory		Europe	congeneric
12	*Duchesnea indica*	**Rosales**	Rosaceae	-	SC	Asia	congeneric
13	*Rubus phoenicolasius*	**Rosales**	Rosaceae	-	SC	Asia	congeneric
13	*Rubus pensilvanicus*	**Rosales**	Rosaceae	apomixis; pseudogamy; facultative		North America	congeneric

Abbreviations: SC, self-compatible; SIC, self-incompatible.

Table S2 Accepted subspecies and synonyms for the 26 species downloaded from GBIF.

Pair ID	Species	TPL ssp accepted	Synonym in TPL	Unresolved or synonym of other species in TPL
1	*Ageratina adenophora*		*Eupatorium adenophorum*	
1	*Mikania micrantha*	-	-	
2	*Chromolaena odorata*		*Eupatorium clematitis*	
			Eupatorium conyzoides	
			Eupatorium divergens	
			Eupatorium klattii	
2	*Eupatorium cannabinum*		*heterophyllum*	
			allaisii	
3	*Erigeron annuus*	*ssp septentrionalis*	*var annuus*	
			var discoideus	
3	*Erigeron canadensis*	*glabrata*	*Conyza canadensis var pusilla*	
		Canadensis	*Conyza parva*	
		Conyza canadensis		
4	*Euphorbia esula*	*tommasiniana*	*androsaemifolia*	x pseudovirgata
		virgultosa	*karoi*	
		waldsteinii	*loreyi*	
			lunulata	
			paludosa	
			salicetorum	
			saratoi	
			tarokoensis	
			Tithymalus uralensis	
4	*Euphorbia cyparissias*	-	-	
5	*Hypericum androsaemum*	-	-	
5	*Hypericum perforatum*		*ssp/var angustifolium*	
			ssp microphyllum/veronese	
6	*Clidemia hirta*	-	-	
6	*Miconia calvescens*	-	-	
7	*Brachiaria brizantha*	-	-	
7	*Brachiaria ruziziensis*	-	-	
8	*Cortaderia jubata*			
8	*Cortaderia selloana*		*argentea; Gynerium argenteum*	
9	*Eragrostis curvula*		*capillifolia*	
			chloromelas	
			huillensis	
			jeffreysii	
			Poa curvula	
9	*Eragrostis superba*		*platystachys*	
10	*Paspalum conjugatum*	-	-	
10	*Paspalum urvillei*	-	-	
11	*Poa pratensis*	*ssp latifolia*	*costata*	
		var. hautismae		ssp/var *angustifolia*
			agassiziensis	
			bourgeaei	
			brintnellii	
			oligeria	
			colpodea; anceps	ssp/var *alpigena* ssp/var *colpodea* = *alpigena*

11	*Poa annua*	*var. annua*	*stenachyra*<br>*var reptans*		subsp *raniglumis*<br>subsp. *supina*<br>subsp. *exilis*<br>subsp. *varia*
12	*Potentilla recta*	*ssp sulphurea*	*hirta*		reptans subsp. recta
12	*Duchesnea indica*	-		-	
13	*Rubus phoenicolasius*				var *strigosus* not found!
13	*Rubus pensilvanicus*	-		-	

Only downloaded synonyms were checked for validity in The Plant List (TPL, http://www.theplantlist.org/). Occurrence data pertaining to unresolved names or synonyms of other species (right-hand column) were discarded.

Table S3 Additional sources for range definition of the 26 study taxa.

pair ID	Species	Region/Country	Source
1	*Ageratina adenophora*	Philippines - invasive	**Muniappan R, Raman A, Reddy GVP. 2009.** *Ageratina adenophora* (Sprengel) King and Robinson (Asteraceae). In: Muniappan R, Reddy GVP, Raman A, eds. *Biological Control of Tropical Weeds using Arthropods*. Cambridge, UK: Cambridge University Press, 63-73.
		South Africa, Zimbabwe, Nigeria - invasive	**Muniappan R, Raman A, Reddy GVP. 2009.** *Ageratina adenophora* (Sprengel) King and Robinson (Asteraceae). In: Muniappan R, Reddy GVP, Raman A, eds. *Biological Control of Tropical Weeds using Arthropods*. Cambridge, UK: Cambridge University Press, 63-73.
1	*Mikania micrantha*	Brunei, Papua, Indonesia and pacific islands - invasive	http://keyserver.lucidcentral.org/weeds/data/03030800-0b07-490a-8d04-0605030c0f01/media/Html/Mikania_micrantha.htm
2	*Chromolaena odorata*	Florida, Mexico - native	**Cruttwell Mc Fadyen R, Skarratt B. 1996**. Potential disribution of *Chromolaena odorata* (siam weed) in Australia, Africa and Oceania. *Agriculture, Ecosystems & Environment* **59**: 89-96.
		Cocos Islands - invasive	http://archive.agric.wa.gov.au/PC_95009.html?s=0
		Cote d'Ivoire, Congo, Brunei, Papua New Guinea, Vietnam, Indonesia - invasive	http://www.cabi.org/isc/datasheet/23248
2	*Eupatorium cannabinum*	Europe - native	**Tutin TG. 1976.** *Flora Europaea*. 4. Plantaginaceae to Compostae (and Rubiaceae). 1st edn. Cambridge, Cambridge University Press.
3	*Erigeron annuus*	Austria - invasive	**Tutin TG. 1976.** *Flora Europaea*. 4. Plantaginaceae to Compostae (and Rubiaceae). 1st edn. Cambridge, Cambridge University Press.
		Finland - invasive	http://www.nobanis.org/
		UK - invasive	http://www.ars-grin.gov/cgi-bin/npgs/html/taxon.pl?104210
		erroneous records for Saudi Arabia, Egypt, New Guinea and Greece deleted	http://taif.tfri.gov.tw/search_spec_image.php?SpcmID=111241&DupID=1&SerilD=1&DetID=223086&l=Eng&INo=P1
3	*Conyza canadensis*	Finland, Ireland, Switzerland, Andorra, Austria - invasive	**Tutin TG. 1976.** *Flora Europaea*. 4. Plantaginaceae to Compostae (and Rubiaceae). 1st edn. Cambridge, Cambridge University Press.

		Afghanistan	http://jacq.nhm-wien.ac.at/djatoka/jacq-viewer/viewer.html?rft_id=w_19560000310&identifiers=w_19560000310
		Peru, Dominican Republic, Netherlands Antilles - invasive	(HEAR), http://www.ars-grin.gov/cgi-bin/npgs/html/taxon.pl?104213
4	*Euphorbia esula*	Brazil - discarded	
		Taiwan - discarded	http://www.efloras.org/browse.aspx?flora_id=100&name_str=Euphorbia+esula&btnSearch=Search
		Madagascar - discarded	http://www.wildmadagascar.org/flora/
		Australia - discarded	Atlas of living Australia, http://www.ala.org.au/
4	*Euphorbia cyparissias*	Scandinavia: non native	**Tutin TG. 1978.** *Flora Europaea*. 2. Rosaceae to Umbelliferae. 1st edn. Cambridge, Cambridge University Press. http://linnaeus.nrm.se/flora/welcome.html
		NZ/Australia	http://biocache.ala.org.au/occurrences/search?q=lsid:urn:lsid:biodiversity.org.au:apni.taxon:429818&start=20#tab_recordsView
5	*Hypericum androsaemum*	Japan - discarded	http://foj.c.u-tokyo.ac.jp/gbif/foj/
5	*Hypericum perforatum*	Central America, Papua New Guinea - invasive	PIER, 2003; GBIF, 2005; USDA-GRIN, 2004
6	*Clidemia hirta*	Tanzania - invasive	**DeWalt SJ, Denslow JS, Ickes K. 2004.** Natural-enemy release facilitates habitat expansion of the invasive tropical shrub *Clidemia hirta*. *Ecology* **85**: 471-483.
		West Indies native	http://www.cabi.org/isc/datasheet/13934
		Indian Ocean Islands - invasive	Global Invasive Species Database; http://indiabiodiversity.org/observation/show/272801
6	*Miconia calvescens*	-	
7	*Brachiaria brizantha*	continental Africa - native	http://www.tropicalforages.info/key/Forages/Media/Html/Brachiaria_brizantha.htm
		Madagascar - invasive	http://www.fao.org/ag/agp/AGPC/doc/Counprof/Madagascar/madagascareng.htm
		Thailand/Asia - introduced	**Nakamanee G, Phaikaew C. 1998.** Seed Production potential of *Brachiaria* species in northeast Thailand. In: Stür WW, ed. *Proceedings of the third regional meeting of the FSP*. Samarinda, Indonesia. CIAT Working Document 188: 155-162. http://ciat-library.ciat.cgiar.org/articulos_ciat/seed_production_155.pdf

7	*Brachiaria ruziziensis*	Thailand/Asia	**Nakamanee G, Phaikaew C. 1998.** Seed Production potential of *Brachiaria* species in northeast Thailand. In: Stür WW, ed. *Proceedings of the third regional meeting of the FSP*. Samarinda, Indonesia. CIAT Working Document 188: 155-162. http://ciat-library.ciat.cgiar.org/articulos_ciat/seed_production_155.pdf
		Kenia	https://www.genesys-pgr.org/acn/t/Brachiaria/ruziziensis)
8	*Cortaderia jubata*	Colombia - distribution extended recently	**Giraldo-Canas D, Mayorga C. 2001.** Nuevos registros de gramíneas (Poaceae) para la flora de Colombia. *Hickenia* **3**: 99-103.
8	*Cortaderia selloana*	Netherlands, Ireland - aliens	
		Bolivia - native	http://www.tropicos.org/Name/25509455?projectid=13
		Colombia - introduced	**García-Ulloa JA, Lastra C, Salas C, Merchán MM. 2005.** Estudios en gramíneas (Poaceae) de Colombia: veinte novedades corológicas. *Caldasia* **27**: 131-145.
		Costa Rica - introduced	http://www.ars-grin.gov/cgi-bin/npgs/html/taxon.pl?11618
9	*Eragrostis curvula*	Turkey - introduced	https://www.genesys-pgr.org/acn/id/415470
9	*Eragrostis superba*	Mexico, Honduras - invasive	http://www.kew.org/data/grasses-db/www/imp04051.htm
		Madagascar - invasive	http://www.fao.org/ag/agp/AGPC/doc/Counprof/Madagascar/madagascareng.htm
		Sao Tome - native	**Figueiredo E, Paiva J, Stévart T, Oliveira F, Smith GF. 2011.** Annotated catalogue of the flowering plants of Sao Tomé and Príncipe. *Bothalia* **41**: 41-82.
10	*Paspalum urvillei*	New Caledonia	http://keyserver.lucidcentral.org/weeds/data/03030800-0b07-490a-8d04-0605030c0f01/media/Html/Paspalum_urvillei.htm
		Mexico - invasive	https://www.genesys-pgr.org/acn/id/113623
		Caribbean, French Guiana, Ecuador - native	http://www.cabi.org/isc/datasheet/109621
10	*Paspalum conjugatum*	Old World and Pacific Islands - introduced	**Beetle AA. 1974**. Sour Paspalum - Tropical Weed or Forage? *Journal of Range Management* **27**: 347-349.
		Caribbean - native	http://ecflora.cavehill.uwi.edu/plantdetails.php?pid=1932&sn=Paspalum+conjugatum&cn=&gh=herb
12	*Poa pratensis*	subsp alpigena - native*	http://plants.usda.gov/core/profile?symbol=POPR
		subsp colpodea; subsp pratensis; subsp irrigata - introduced/both native and introduced*	http://plants.usda.gov/core/profile?symbol=POPR

		Falkland Islands - invasive	http://www.issg.org/database/species/distribution_detail.asp?si=1419&di=46550&pc=*
		Nepal - introduced	http://www.efloras.org/florataxon.aspx?flora_id=110&taxon_id=242424491
		Canada - introduced (native in remote mountain regions)	http://www.fs.fed.us/database/feis/plants/graminoid/poapra/all.html
12	*Poa annua*	South America - introduced	http://www.iucnredlist.org/details/168729/0
		Japan - introduced	http://www.nies.go.jp/biodiversity/invasive/resources/listen_poaceae.html
		China - native weed	http://www.efloras.org/flora_page.aspx?flora_id=2
		Taiwan - invasive	http://www.efloras.org/flora_page.aspx?flora_id=102
13	*Duchesnea indica*	-	
13	*Potentilla recta*	Finland, Germany, Switzerland - introduced	**Kurtto A, Jalas J. 2004.** *Atlas florae Europaeae: distribution of vascular plants in Europe*. 13. Rosaceae (Spiraea to Fragaria, excl. Rubus). Helsinki, Akat. Kirjakauppa.
		Andorra, Montenegro - native	**Kurtto A, Jalas J. 2004.** *Atlas florae Europaeae: distribution of vascular plants in Europe*. 13. Rosaceae (Spiraea to Fragaria, excl. Rubus). Helsinki, Akat. Kirjakauppa.
		Argentina - discarded	no sources found
		Greenland - discarded	no sources found
14	*Rubus phoenicolasius*	-	
14	*Rubus pensilvanicus*	-	

Range classification was based on the GloNAF-dataset, however, occurrences not listed in the GloNAF database or of doubtable reliability were checked by literature and database screens. Databases were accessed in May and June 2014.

* native and invasive status of *Poa pratensis* in North America: the status of *P. pratensis* in North America is unclear, possibly subspecies *alpigena* is native while the other subspecies may be mostly introduced with potential for native population in remote montane areas. As *P. pratensis* ssp. *alpigena* was excluded from our dataset (it is a synonym of the species *P. alpigena* (http://www.theplantlist.org/tpl1.1/record/kew-435078, accessed 26.11.2014)) and most of the North American occurrences pertained to *P. pratensis* ssp. *pratensis*, which is considered invasive in most of the US and (http://plants.usda.gov/core/profile?symbol=POPRP2) invasive and native in Canada, the North American occurrences were treated as invasive range.

Table S4 Additional occurrences searched for nine species.

Species	Addition and specification of occurrences
Hypericum perforatum	points in California specified from http://www.calflora.org/cgi-bin/species_query.cgi?where-taxon=Hypericum+perforatum [accessed April 2014]
Ageratina adenophora	Californian points specified by http://www.calflora.org/cgi-bin/species_query.cgi?where-calrecnum=117 [accessed April 2014]
Miconia calvescens	Australia's Virtual Herbarium (www.chah.gov.au/avh) [accessed April 2014]
	Polynesia - based on records in HEAR (http://www.hear.org/pier/species/miconia_calvescens.htm) [accessed April 2014]
Cortaderia jubata	points in California specified via http://www.calflora.org/cgi-bin/species_query.cgi?where-calrecnum=2394 [accessed April 2014]
Cortaderia selloana	points in California specified via http://www.calflora.org/cgi-bin/species_query.cgi?where-taxon=Cortaderia+selloana [accessed April 2014]
Eragrostis curvula	points in California specified via http://www.calflora.org/cgi-bin/species_query.cgi?where-calrecnum=9905 [accessed April 2014]
Paspalum urvillei	points in California specified via http://www.calflora.org/cgi-bin/species_query.cgi?where-taxon=Paspalum+urvillei [accessed April 2014]
Poa pratensis	California points specified via http://www.calflora.org/cgi-bin/species_query.cgi?where-taxon=Poa+pratensis [accessed April 2014]
Poa annua	California points specified via http://www.calflora.org/cgi-bin/species_query.cgi?where-taxon=Poa+annua [accessed April 2014]

For all other species, occurrences are based on GBIF-records only. Occurrences for the above species were added as i) occurrences in the introduced range were underrepresented (*Miconia calvescens*) or as ii) GBIF-occurrences in California were highly imprecise (coordinates without decimal values).

Table S5 Number of occurrences in the alien and native range of the 26 species, respectively, and mean residence times (in years) in a representative subset of the regions comprising the total alien range.

Species	Alien range	Native range	Mean residence time
Ageratina adenophora	1586	49	88 (n = 9)
Mikania micrantha	112	862	18 (n = 2)
Chromolaena odorata	177	583	37 (n = 2)
Eupatorium cannabinum	49	36517	89 (n = 2)
Erigeron annuus	5609	465	153 (n = 26)
Erigeron canadensis	28803	1268	201 (n = 33)
Euphorbia esula	1545	3892	159 (n = 8)
Euphorbia cyparissias	2353	8318	138 (n = 10)
Hypericum androsaemum	97	4266	180 (n = 4)
Hypericum perforatum	2104	47302	140 (n = 7)
Clidemia hirta	50	661	52 (n = 3)
Miconia calvescens	34	328	41 (n = 3)
Brachiaria brizantha	73	421	40 (n = 3)
Brachiaria ruziziensis	34	33	52 (n = 2)
Cortaderia jubata	44	67	61 (n = 2)
Cortaderia selloana	788	42	60 (n = 11)
Eragrostis curvula	1815	937	83 (n = 11)
Eragrostis superba	68	417	63 (n = 2)
Paspalum urvillei	360	906	94 (n = 9)
Paspalum conjugatum	1076	147	77 (n = 5)
Poa pratensis	2457	105795	114 (n = 8)
Poa annua	2322	94467	146 (n = 9)
Potentilla recta	3298	278	131 (n = 10)
Duchesnea indica	1649	456	95 (n = 24)
Rubus pensilvanicus	49	124	112 (n = 1)
Rubus phoenicolasius	177	121	74 (n = 8)

Table S6 Additional sources for time since introduction (residence times)

All other dates of introduction have been taken from the GloNAF database.

Species	Country	Year	Source
Paspalum urvillei	USA	1921	http://plants.usda.gov/java/reference?symbol=PAUR2 [accessed June 2015]
Rubus pensilvanicus	California	1903	http://ucjeps.berkeley.edu/cgi-bin/get_consort.pl?dups=UCR256076%20SD205056%20SD221287%20UCR116681%20RSA733010%20SD183091%20JEPS47295%20JEPS47286%20UCR65203%20UCR79497%20RSA61874%20UCR75654%20UCR17465%20SBBG38435%20SBBG90463%20UCR3414%20CAS343921%20CAS453224%20UCR55875%20UCR25376%20UCR99342%20UCR95318%20UCR111587%20UCR4188%20UCR4119%20UCR97807%20UCR48584%20CAS593508%20CAS866560%20CAS538945%20UCR97837%20RSA614973%20UC61019%20UCD77977%20UC1281058%20CAS878095%20UCD113805%20UCD91414%20CAS866603%20CAS878096%20UCD89070%20JEPS117304%20CHSC88086%20RSA699538%20CHSC44231%20CHSC50341%20CHSC65432%20CHSC65434%20CHSC65431%20CHSC65433%20BLMAR240%20CHSC51370 [accessed June 2015]
Miconia calvescens	Hawaii	1960	http://www.issg.org/database/species/ecology.asp?si=2 [accessed June 2015]
Miconia calvescens	French Polynesia	1992	http://www.issg.org/database/species/ecology.asp?si=2 [accessed June 2015]
Miconia calvescens	Australia	1970	https://www.daf.qld.gov.au/__data/assets/pdf_file/0005/58082/IPA-Miconia-Risk-Assessment.pdf [accessed June 2015]
Brachiaria brizantha	Sri Lanka	1955	http://www.fao.org/wairdocs/ilri/x5491e/x5491e0c.htm [accessed June 2015]
Brachiaria ruziziensis	Brazil	1960	Kumble, Vrinda (1996). *Brachiaria: Biology, Agronomy, and Improvement*. CIAT.
Brachiaria ruziziensis	Australia	1966	https://books.google.at/books?id=dMF6QpfVdjMC&pg=PA258&lpg=PA258&dq=Brachiaria+ruziziensis+date+of+introduction&source=bl&ots=_7yZOx68Y4&sig=cj8glpXQm2BjNbqhaBXkdbs5F7E&hl=de&sa=X&ved=0CDsQ6AEwA2oVChMImtyep8eHxgIVwiVyCh3XZABh#v=onepage&q=ruziziensis&f=false [accessed June 2015]
Brachiaria brizantha	Brazil	1984	https://books.google.at/books?id=dMF6QpfVdjMC&pg=PA258&lpg=PA258&dq=Brachiaria+ruziziensis+date+of+introduction&source=bl&ots=_7yZOx68Y4&sig=cj8glpXQm2BjNbqhaBXkdbs5F7E&hl=de&sa=X&ved=0CDsQ6AEwA2oVChMImtyep8eHxgIVwiVyCh3XZABh#v=onepage&q=ruziziensis&f=false [accessed June 2015]
Brachiaria brizantha	Colombia	1987	https://books.google.at/books?id=dMF6QpfVdjMC&pg=PA258&lpg=PA258&dq=Brachiaria+ruziziensis+date+of+introduction&source=bl&ots=_7yZOx68Y4&sig=cj8glpXQm2BjNbqhaBXkdbs5F7E&hl=de&sa=X&ved=0CDsQ6AEwA2oVChMImtyep8eHxgIVwiVyCh3XZABh#v=onepage&q=ruziziensis&f=false [accessed June 2015]
Eragrostis superba	Australia	1933	http://biocache.ala.org.au/occurrence/search?q=lsid%3Aurn%3Alsid%3Abiodiversity.org.au%3Aapni.taxon%3A322311 [accessed June 2015]
Eragrostis curvula	Australia	1914	http://biocache.ala.org.au/occurrences/search?q=matched_name_children%3A%22Eragrostis+curvula%22+occurrence_date%3A[*+TO+1920-01-01T00%3A00%3A00Z] [accessed June 2015]
Duchesnea indica	USA	1901	http://plants.usda.gov/java/reference?symbol=DUIN2 [accessed June 2015]
Rubus phoenicolasius	USA	1940	http://plants.usda.gov/java/reference?symbol=RUPH [accessed June 2015]

Fig. S1 Maps of the native and alien ranges of the 26 study species based on GloNAF and occurrence records derived from GBIF. Native ranges are depicted in yellow, alien ranges in red, black points indicate occurrences.

Pair 1

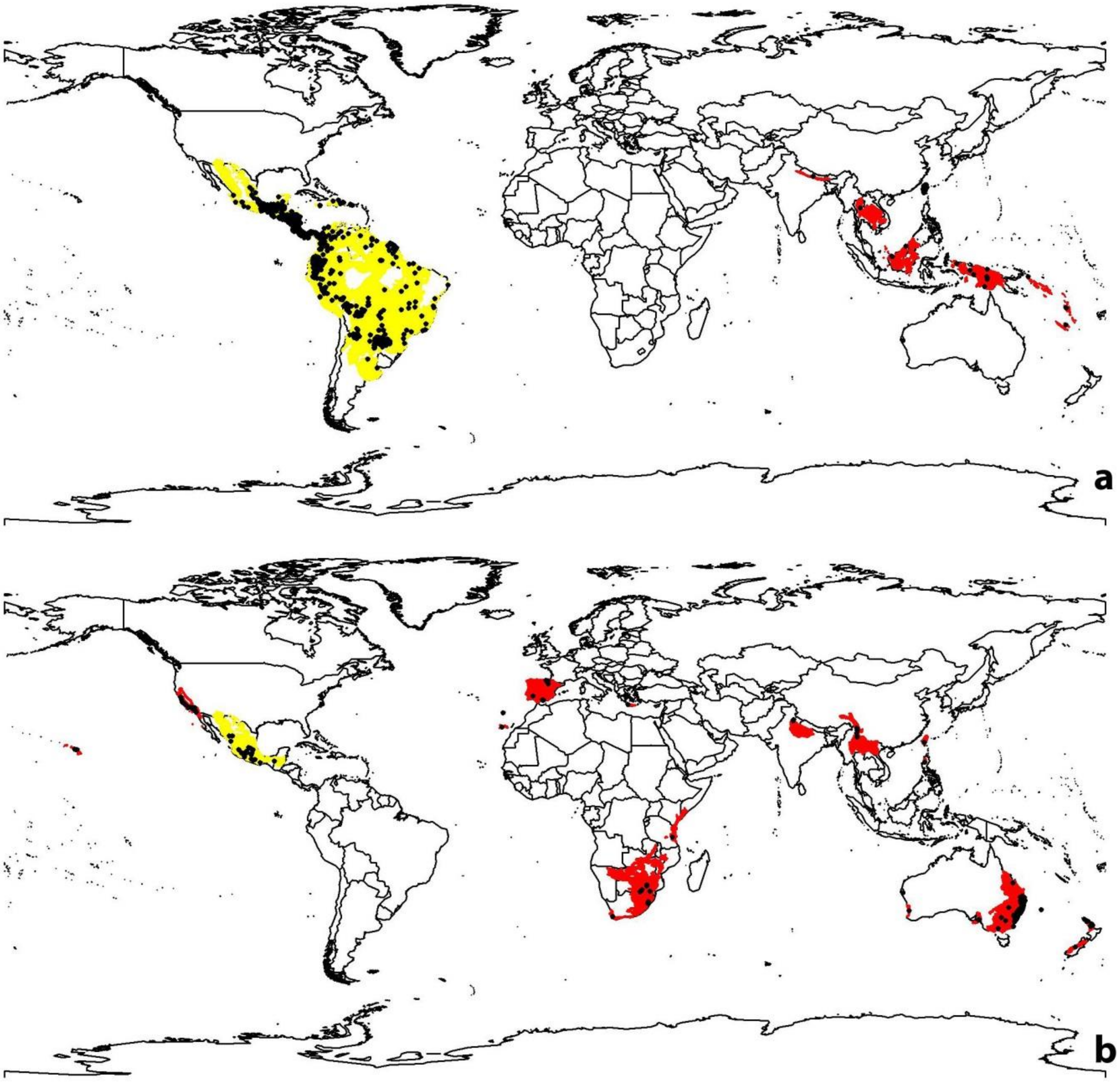

a – *Mikania micrantha*
b – *Ageratina adenophora*

Pair 2

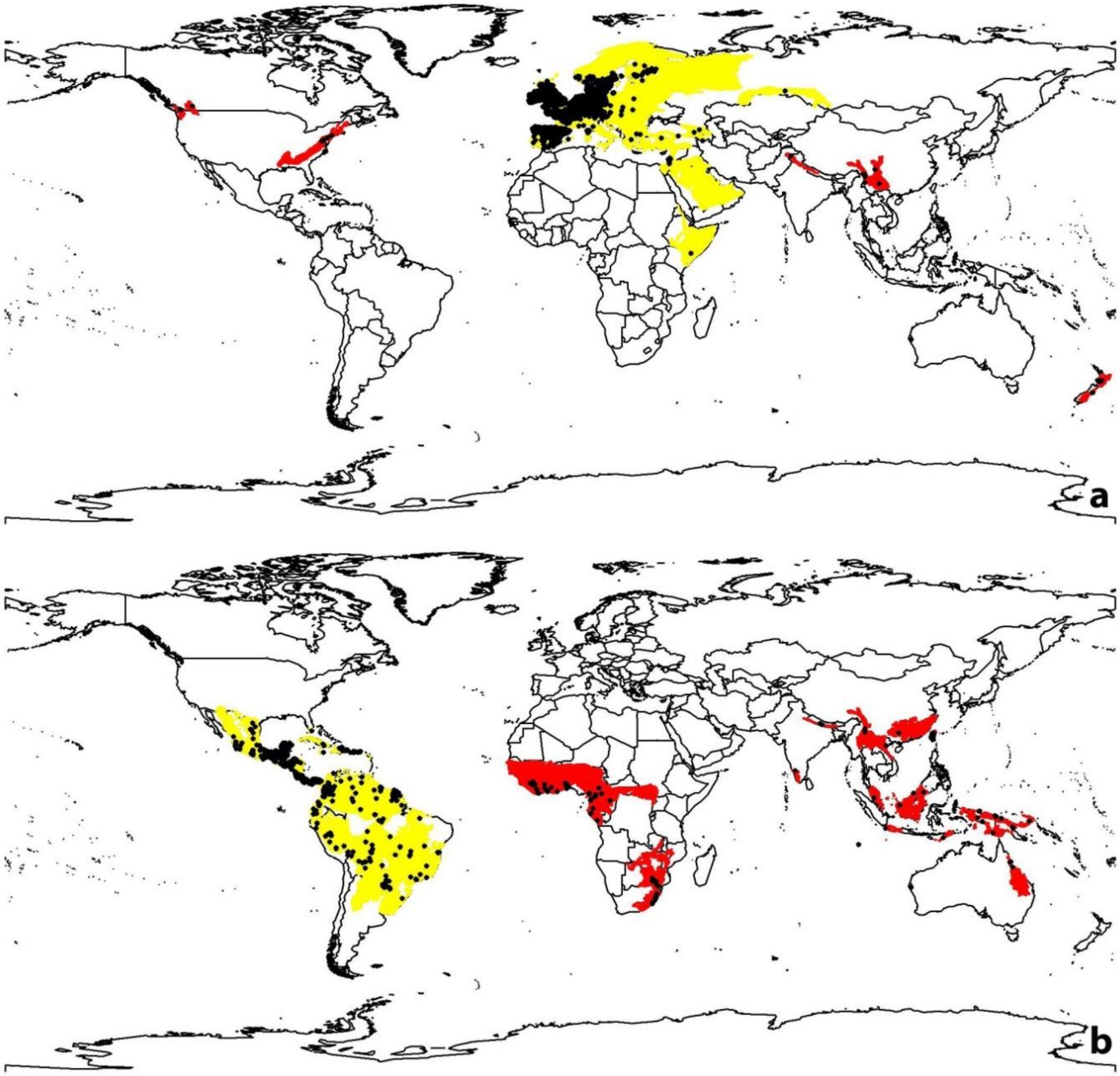

a – *Eupatorium cannabinum*
b – *Chromolaena odorata*

Pair 3

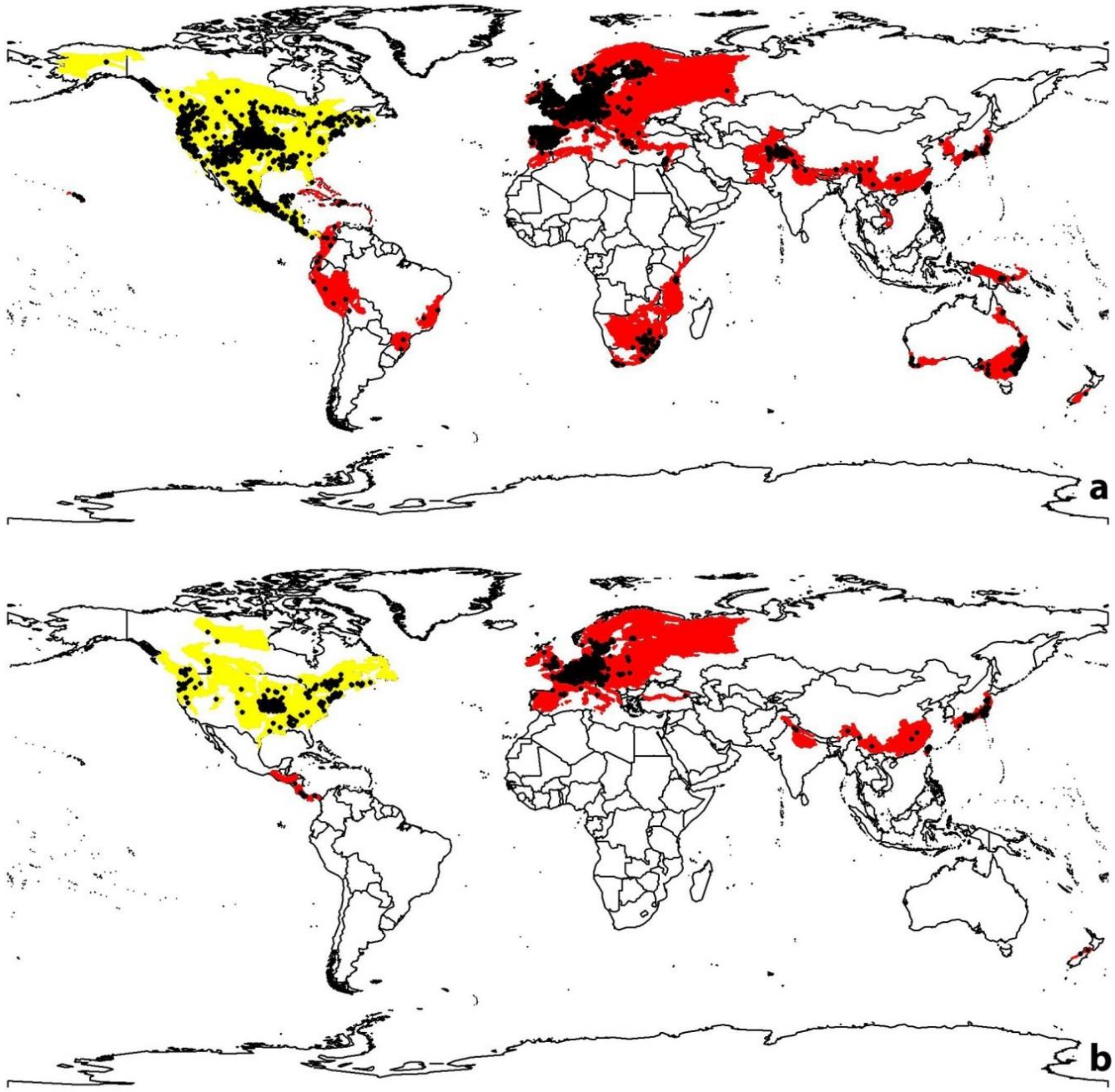

a – *Erigeron canadensis*
b – *Erigeron annuus*

Pair 4

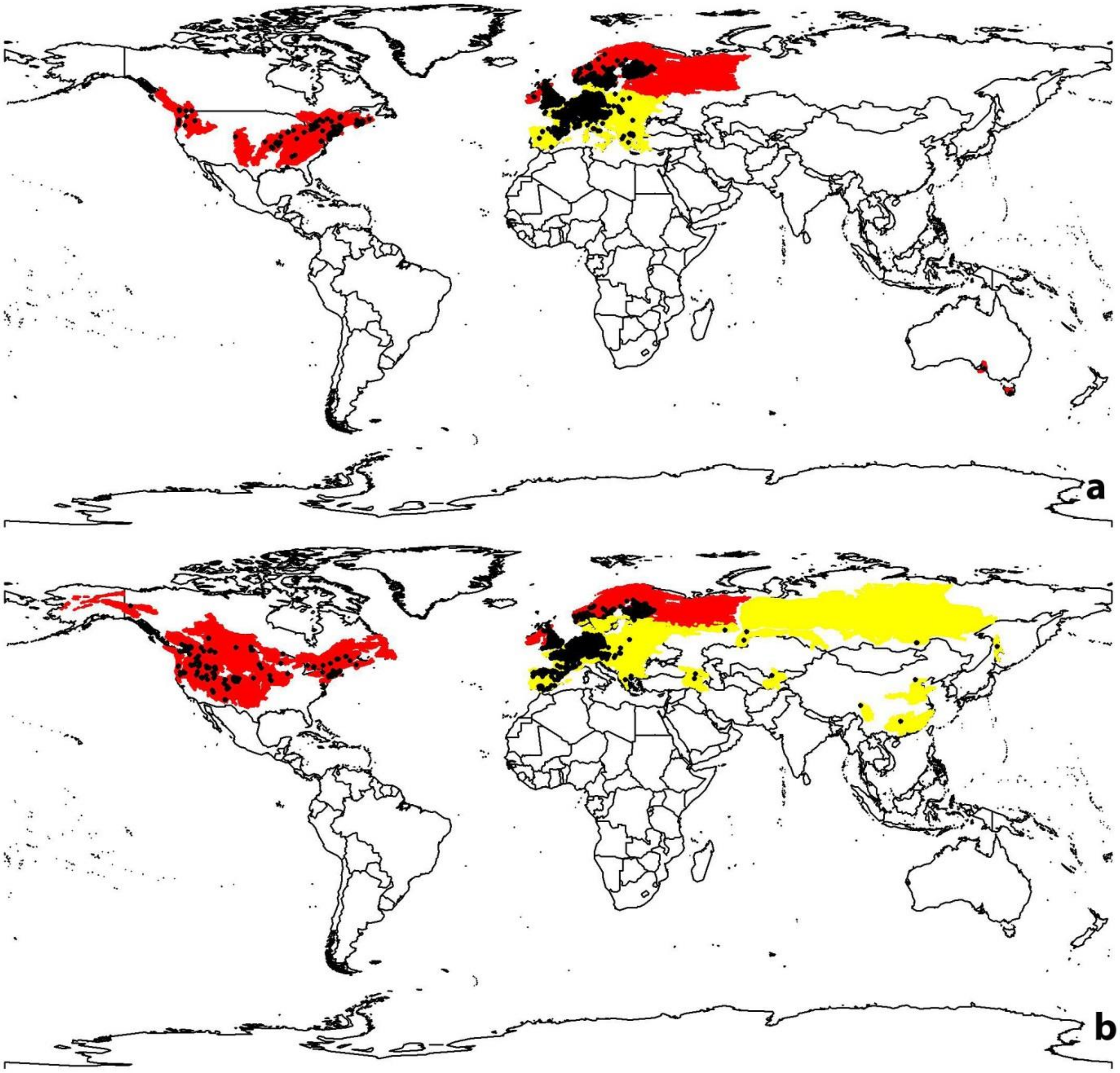

a – *Euphorbia cyparissias*
b – *Euphorbia esula*

Distributions in Northern Europe are considered archaeophytic in the Atlas Flora Europaea and have been treated as alien ranges here.

Pair 5

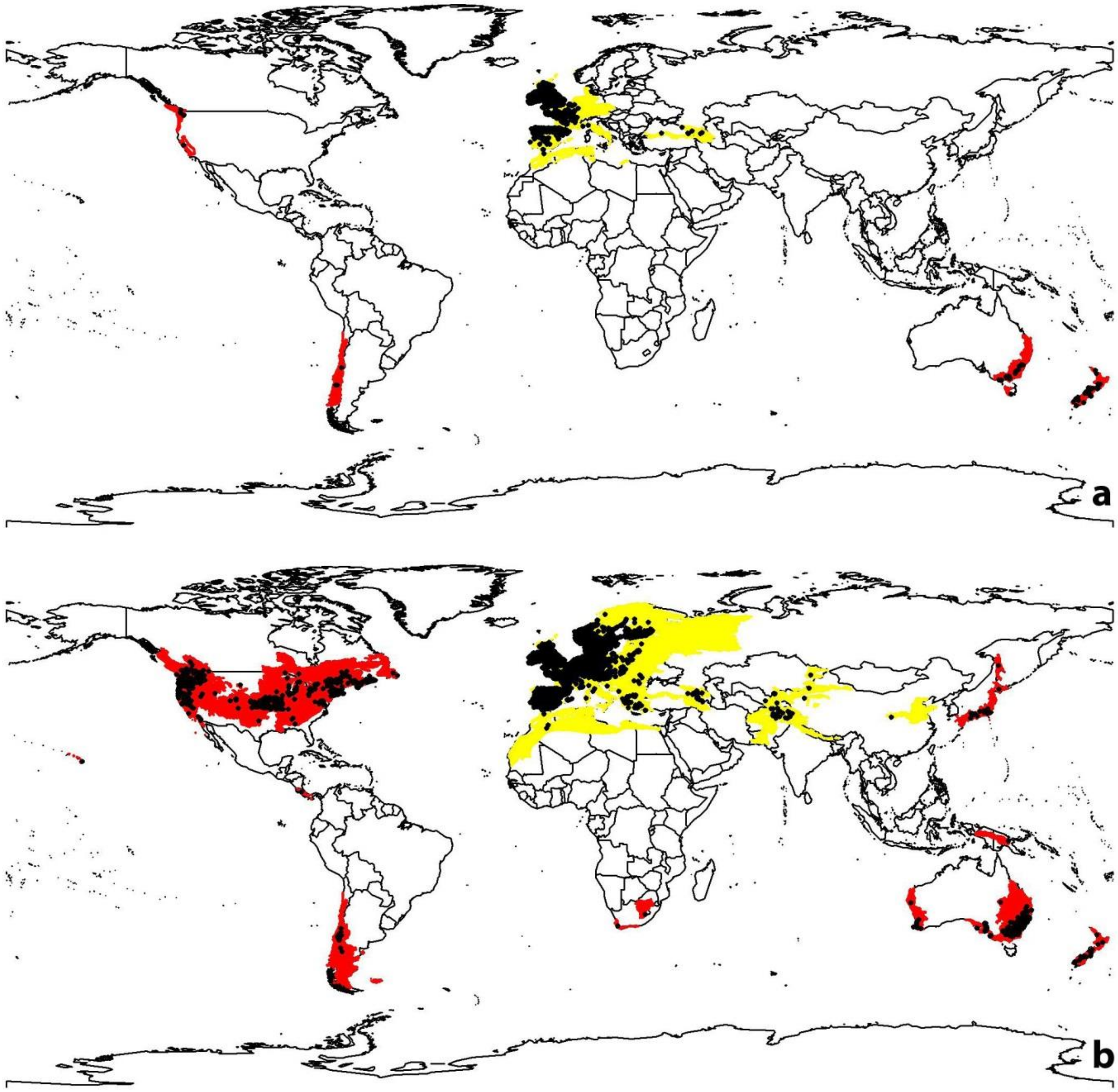

a – *Hypericum androsaemum*
b – *Hypericum perforatum*

Pair 6

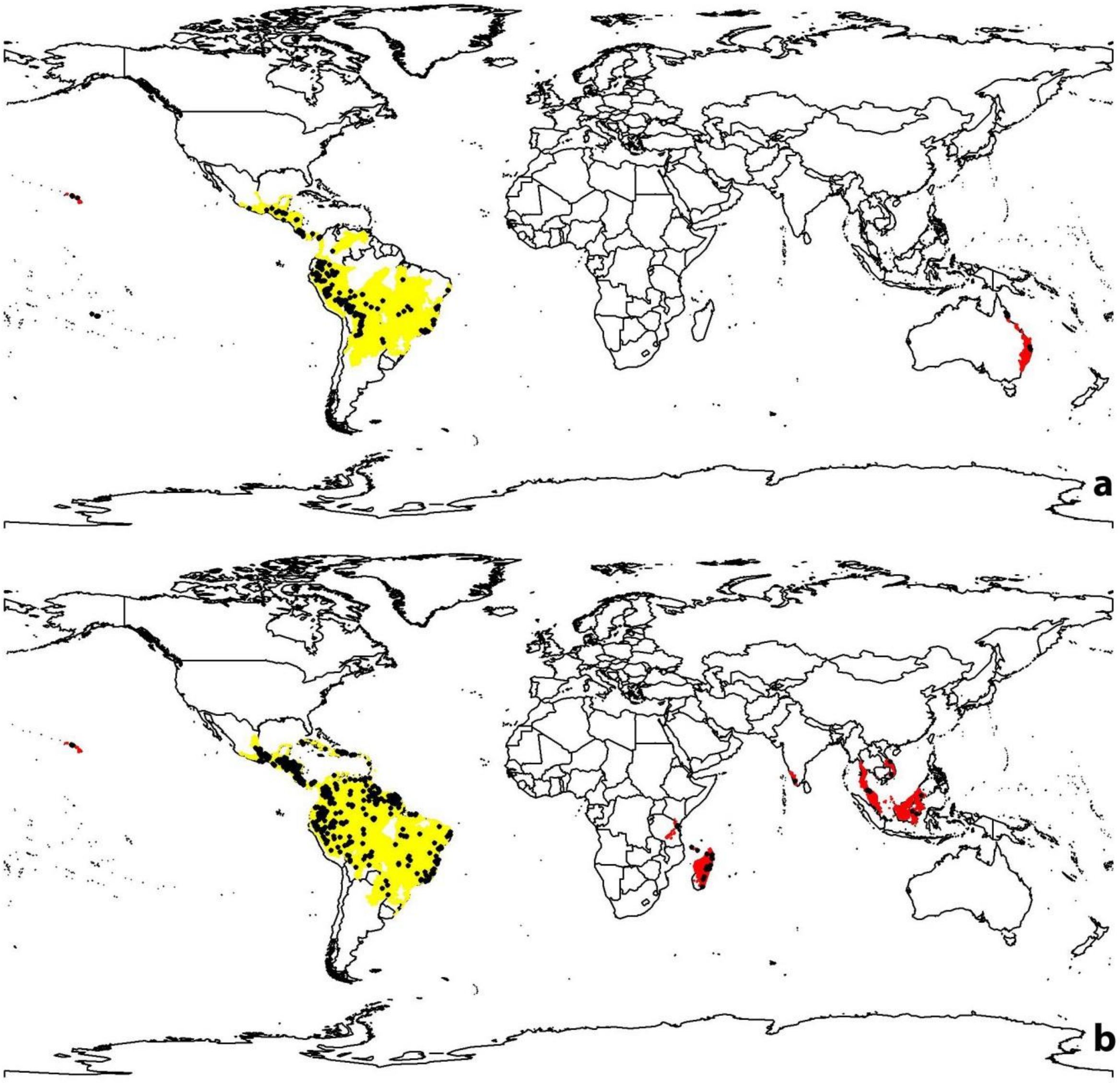

a – *Miconia calvescens*
b – *Clidemia hirta*

Pair 7

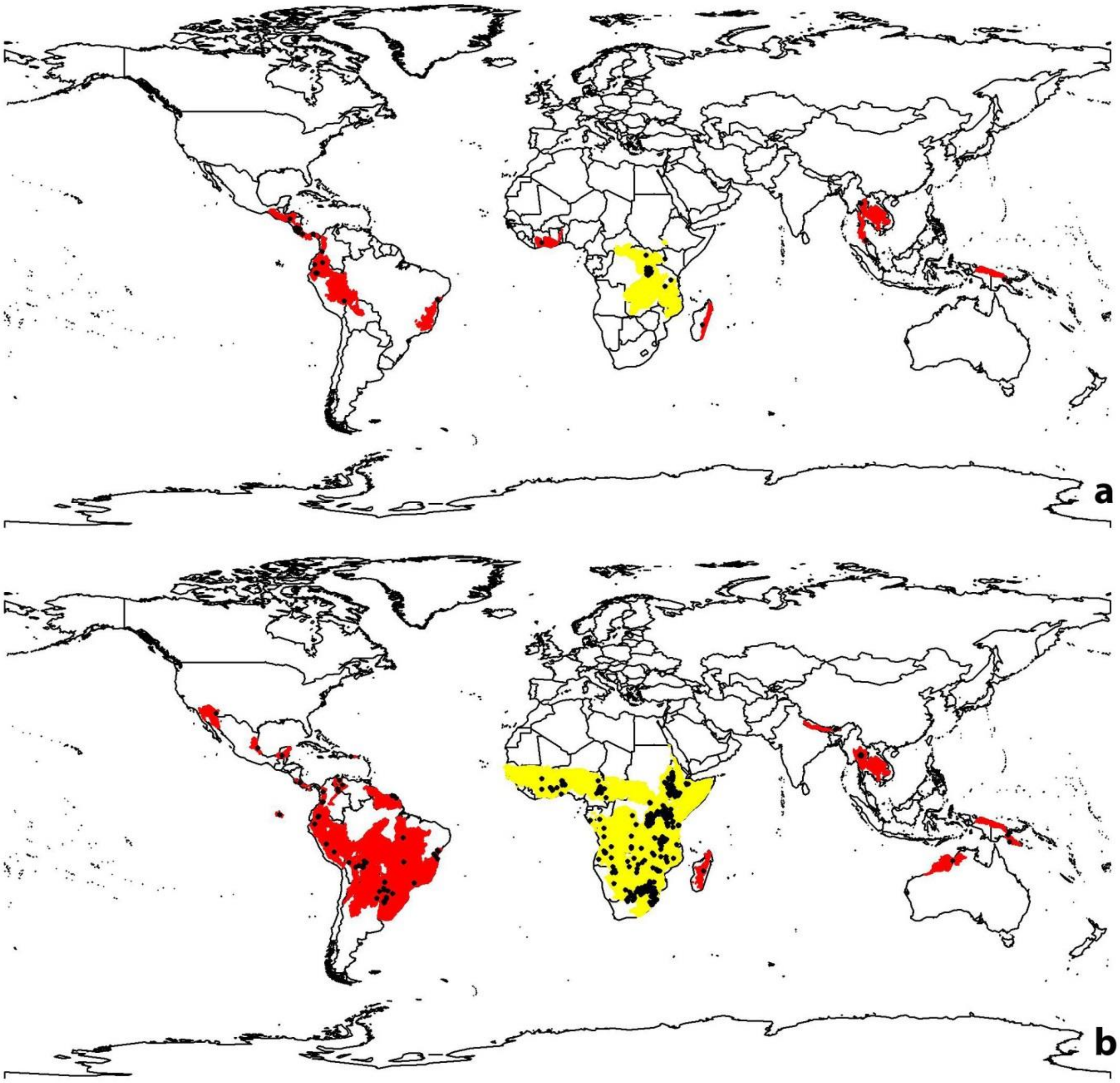

a – *Brachiaria ruziziensis*
b – *Brachiaria brizantha*

Pair 8

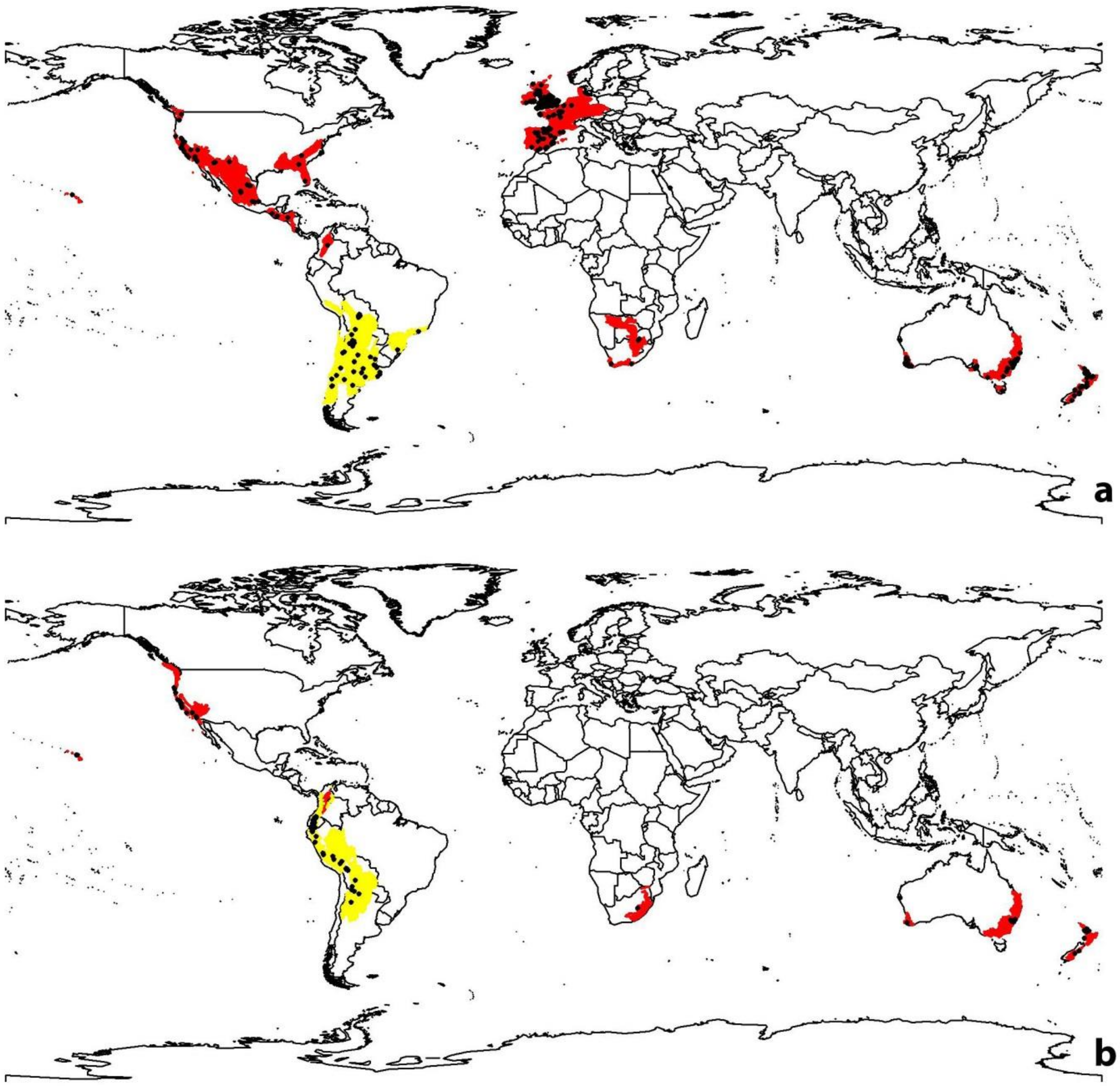

a – *Cortaderia selloana*
b – *Cortaderia jubata*

Pair 9

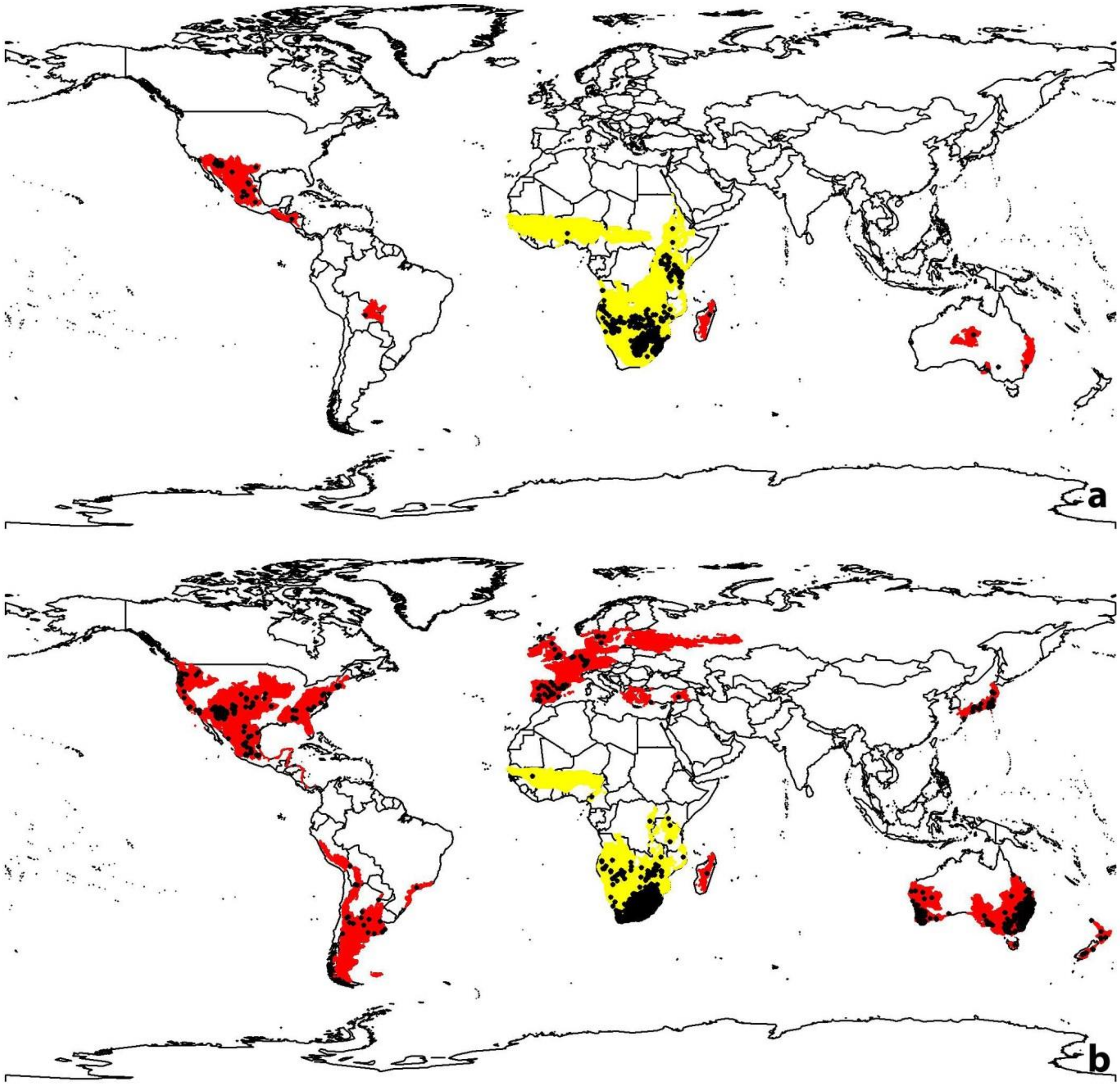

a – *Eragrostis superba*
b – *Eragrostis curvula*

Pair 10

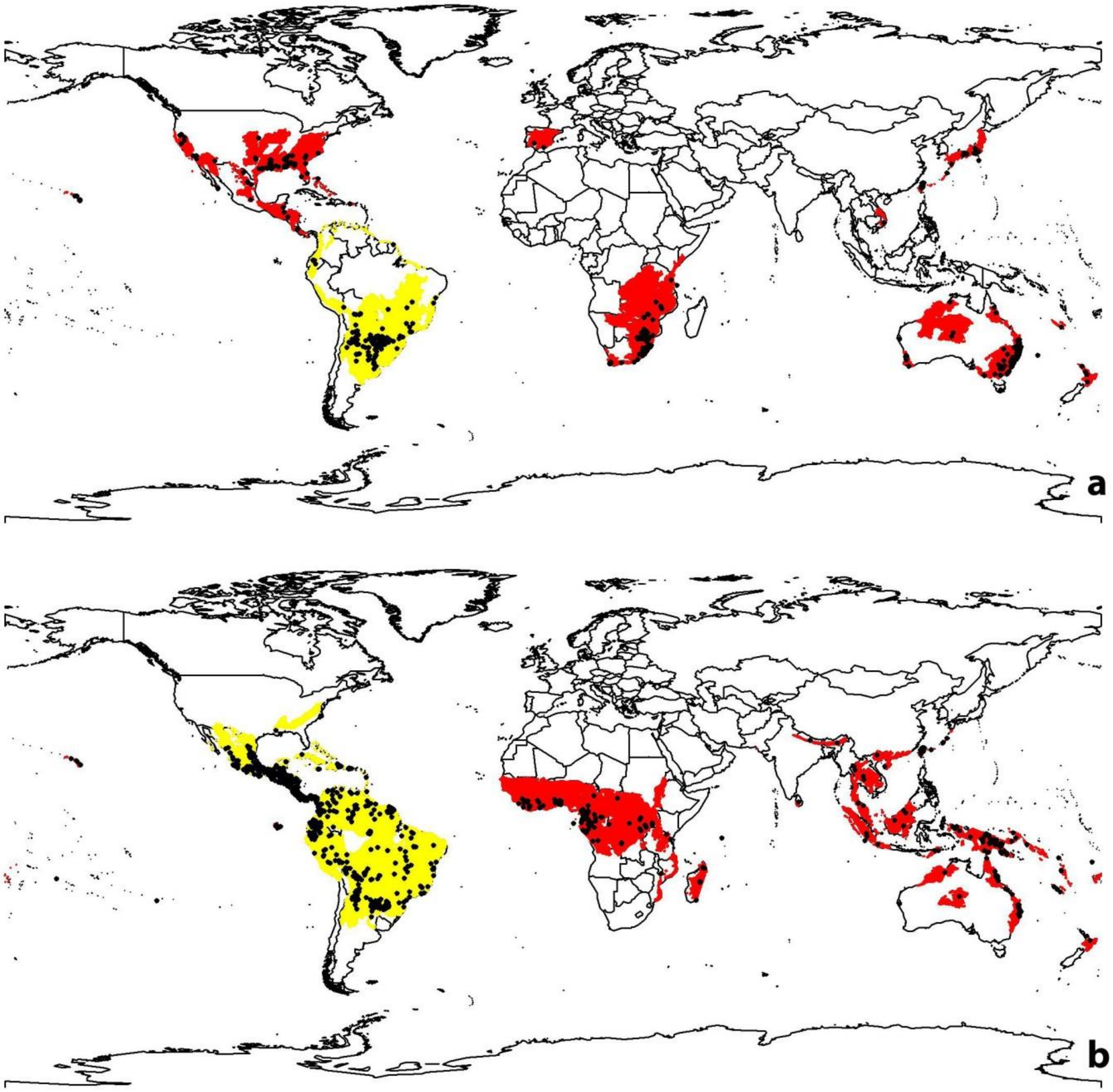

a – *Paspalum urvillei*
b – *Paspalum conjugatum*

Pair 11

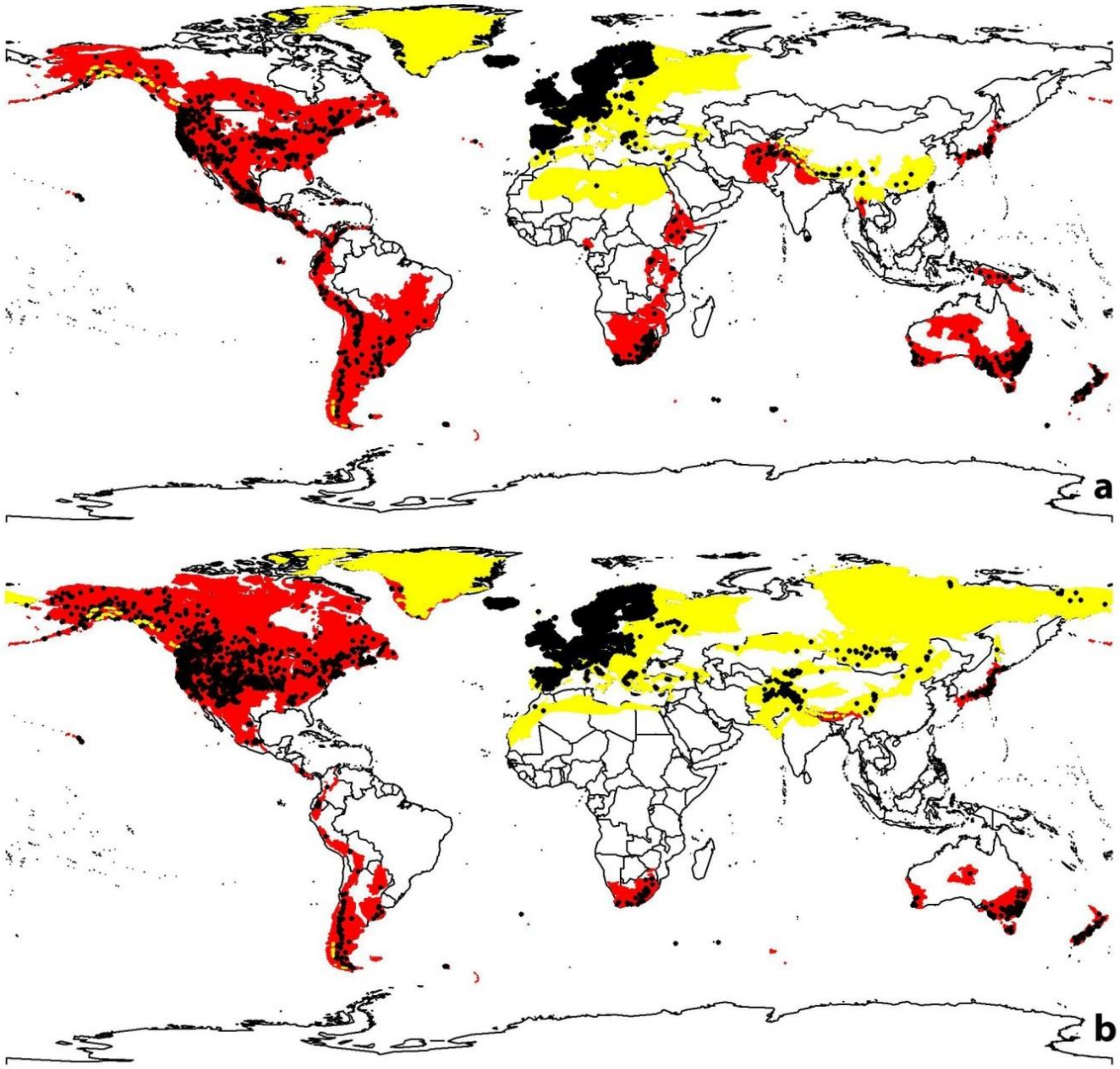

a – *Poa annua*
b – *Poa pratensis*

See comments on the distribution of these two species in Table S3. An overlap of ranges (e.g. in North and South America) is the result of the use of the WWF biogeographical regions which group together biogeographically similar habitats.

Pair 12

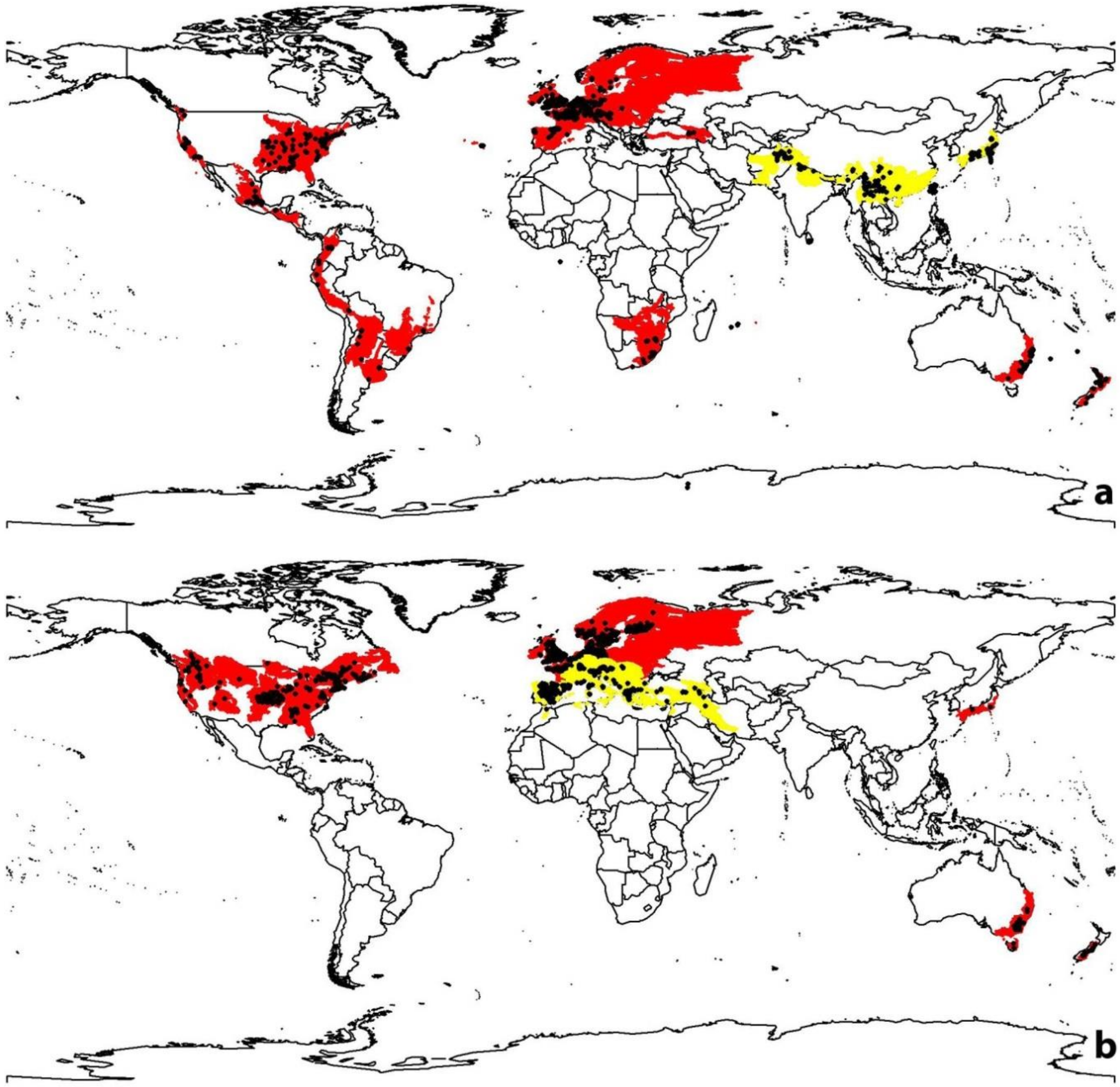

a – *Duchesnea indica*
b – *Potentilla recta*

Pair 13

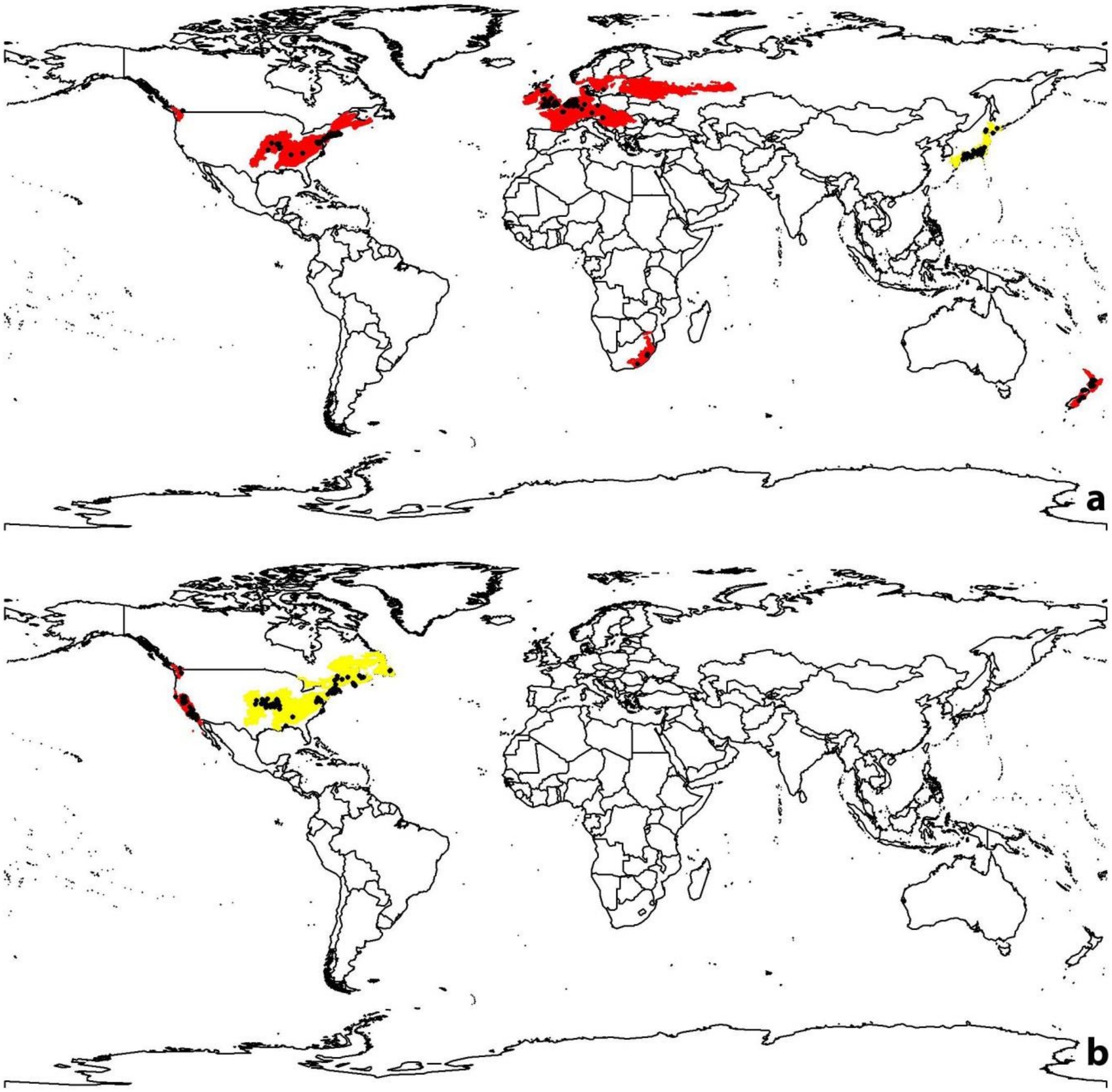

a – *Rubus phoenicolasius*
b – *Rubus pensilvanicus*

Notes S1 Tests of equivalency and similarity of native and alien niches

According to the niche similarity test, niches were more similar than expected by chance in 18 species (Table S7). Twelve species only had niches in one range that were more similar than if projected in the other range (only invasive niche more similar: *Eupatorium cannabinum, Erigeron annuus, Euphorbia esula, Euphorbia cyparissias, Hypericum perforatum, Cortaderia jubata;* only native niche more similar: *Ageratina adenophora*, *Brachiaria brizantha*, *Brachiaria ruizizensis, Cortaderia jubata*, *Eragrostis superba*, *Paspalum urvillei*). Note that a non-significant result of the niche similarity test does not automatically mean niche divergence (Warren *et al.*, 2008).

References

Warren DL, Glor RE, Turelli M. 2008. Environmental niche equivalency versus conservatism: quantitative approaches to niche evolution. *Evolution* **62:** 2868–2883.

Table S7 Results from the niche equivalency and the two niche similarity tests which test for significant niche overlap (D) between the native and invasive range for each species Significant *P*-values (*P* < 0.05) are shown in bold.

Species	D	Equivalency	Similarity alien -> native	Similarity native -> alien
Ageratina adenophora	0.234	**0.0198**	0.4158	**0.0198**
Mikania micrantha	0.1751	**0.0198**	0.703	0.396
Chromolaena odorata	0.3531	**0.0198**	**0.0099**	**0.0099**
Eupatorium cannabinum	0.3121	**0.0198**	**0.0297**	0.1683
Erigeron annuus	0.2199	**0.0198**	**0.0099**	0.1287
Erigeroncanadensis	0.1926	**0.0198**	0.1188	0.1386
Euphorbia esula	0.2576	**0.0198**	**0.0396**	0.0594
Euphorbia cyparissias	0.4368	**0.0198**	**0.0396**	0.198
Hypericum perforatum	0.3459	**0.0198**	**0.0297**	0.1584
Hypericum androsaemum	0.4275	**0.0198**	0.0891	0.099
Clidemia hirta	0.1959	**0.0198**	0.2772	0.0693
Miconia calvescens	0.1948	**0.0198**	**0.0198**	**0.0297**
Brachiaria brizantha	0.1398	**0.0198**	0.505	**0.0297**
Brachiaria ruziziensis	0.0423	**0.0198**	0.4554	**0.0297**
Cortaderia jubata	0.3368	**0.0198**	0.1485	**0.0297**
Cortaderia selloana	0.3156	**0.0198**	**0.0099**	0.4752
Eragrostis curvula	0.5107	**0.0198**	**0.0297**	**0.0297**
Eragrostis superba	0.2379	**0.0198**	0.4158	**0.0099**
Paspalum conjugatum	0.317	**0.0198**	**0.0099**	**0.0099**
Paspalum urvillei	0.3329	**0.0198**	0.1485	**0.0099**
Poa pratensis	0.3649	**0.0198**	**0.0198**	**0.0495**
Poa annua	0.3208	**0.0198**	**0.0198**	**0.0198**
Potentilla recta	0.3356	**0.0198**	0.1485	0.1386
Duchesnea indica	0.3025	**0.0198**	0.0594	0.1089
Rubus pensilvanicus	0.0026	**0.0198**	1	1
Rubus phoenicolasius	0.0336	**0.0198**	0.9505	0.8218

Notes S2 Results of test on expansion/unfilling using analogous or full climatic spaces, respectively

Calculating niche expansion and unfilling based on either the full climatic space available in both native and introduced ranges or only that part of the alien range's climatic space that is also represented in the native range (= analogous climates) did not change the results pronouncedly (Table S8). In general, including the full climatic space resulted in 0.5-4% more niche expansion or unfilling, respectively, and levels of niche expansion higher than 10% were observed in eight while levels of unfilling higher than 10% were observed in seven out of 26 species. In comparison, when using analogous climates only, seven species expanded

more than 10% into novel niche space and eight species unfilled more than 10% of the native niche space.

Table S8 Proportion of novel niche space occupied in the alien range (Expansion) and proportion of native niche space which is not occupied in the alien range (Unfilling) calculated by including only **analogous climates** of the alien range or the **full climatic space** of both range parts. Values larger than 10% of expansion or unfilling are given in bold.

	**Analogous climates**		**Full climatic space**	
**Species**	**Expansion**	**Unfilling**	**Expansion**	**Unfilling**
Ageratina adenophora	**0.3578**	0.0906	**0.3853**	**0.1031**
Mikania micrantha	0.0254	**0.1574**	0.0333	**0.1917**
Chromolaena odorata	0.0376	0.037	0.0447	0.0393
Eupatorium cannabinum	**0.1603**	**0.1703**	**0.1623**	**0.1724**
Erigeron annuus	0.0189	**0.6575**	0.0236	**0.667**
Erigeroncanadensis	0.002	**0.3579**	0.0022	**0.3698**
Euphorbia esula	**0.4533**	0.0091	**0.4577**	0.0248
Euphorbia cyparissias	**0.2675**	0.0221	**0.3102**	0.0236
Hypericum perforatum	**0.1936**	0.0144	**0.1998**	0.012
Hypericum androsaemum	0.0909	0.0444	**0.1213**	0.0431
Clidemia hirta	0.0054	**0.2824**	0.0055	**0.3006**
Miconia calvescens	0.0003	**0.3523**	0.0002	**0.4191**
Brachiaria brizantha	**0.1482**	**0.2303**	**0.2519**	**0.2189**
Brachiaria ruziziensis	**0.684**	**0.322**	**0.8148**	**0.351**
Cortaderia jubata	0.0848	**0.5112**	0.0848	**0.5366**
Cortaderia selloana	**0.4376**	**0.2267**	**0.4407**	**0.2273**
Eragrostis curvula	0.0271	0.0044	**0.1177**	0.0044
Eragrostis superba	**0.1469**	**0.3589**	**0.19**	**0.3603**
Paspalum conjugatum	0.0269	0.0572	0.0325	0.0658
Paspalum urvillei	**0.1245**	0.0259	**0.1267**	0.0268
Poa pratensis	**0.162**	0.0015	**0.1642**	0.0026
Poa annua	**0.2372**	0.0019	**0.2822**	0.0019
Potentilla recta	0.0919	**0.1803**	**0.105**	**0.1792**
Duchesnea indica	0.0165	**0.1327**	0.0198	**0.1338**
Rubus pensilvanicus	**0.967**	**0.9737**	**0.9954**	**0.9953**
Rubus phoenicolasius	**0.8253**	**0.8864**	**0.9404**	**0.911**

Notes S3 Effects of continent of origin on niche dynamics

We evaluated the effects of the continent of origin on the calculated niche change metrics by means of Linear Mixed Effects Models (LMMs). Continent of origin was used as a fixed effect in the models and species pair ID as a random effects grouping factor. The results demonstrate that that species with their native ranges in either Asia or North America, had undergone a significantly stronger change in niche optimum position (along PCA-axis 2) than African species (Asia: *t*-value 2.441, df = 24, *P* = 0.02; North America: *t*-value 2.297, df = 24, *P* = 0.03). These results do not indicate a systematic bias of uneven GBIF sampling densities on our results because both an intensively sampled continent (North America) and a partly sparsely sampled continent (Asia) are contrasted with another partly sparsely sampled continent (Africa). Species with a Eurasian native distribution had further broadened their niche significantly more than compared to African species (PCA-axis 2, *t*-value = 2.633, df = 24, *P* = 0.01). Similarly, this is hardly explainable by differential GBIF data densities because Europe has extremely high sampling density and Africa an at least partly low one – so that niche broadening should rather be expected for African species if lack of sampling in the native region is the relevant factor. There was no effect of the continent of origin on niche expansion or unfilling.
